# Risk stratification in adult and pediatric pulmonary arterial hypertension: A systematic review

**DOI:** 10.3389/fcvm.2022.1035453

**Published:** 2022-11-10

**Authors:** Chantal Lokhorst, Sjoukje van der Werf, Rolf M. F. Berger, Johannes M. Douwes

**Affiliations:** ^1^Department of Pediatric Cardiology, Center for Congenital Heart Diseases, Beatrix Children’s Hospital, University Medical Center Groningen, University of Groningen, Groningen, Netherlands; ^2^Central Medical Library, University Medical Center Groningen, University of Groningen, Groningen, Netherlands

**Keywords:** pulmonary arterial hypertension, pediatric pulmonary hypertension, risk stratification, risk assessment, survival, outcome, prognosis, children

## Abstract

**Introduction:**

Currently, risk stratification is the cornerstone of determining treatment strategy for patients with pulmonary arterial hypertension (PAH). Since the 2015 European Society of Cardiology/European Respiratory Society (ESC/ERS) guidelines for the diagnosis and treatment of pulmonary hypertension recommended risk assessment, the number of studies reporting risk stratification has considerably increased. This systematic review aims to report and compare the variables and prognostic value of the various risk stratification models for outcome prediction in adult and pediatric PAH.

**Methods:**

A systematic search with terms related to PAH, pediatric pulmonary hypertension, and risk stratification was performed through databases PubMed, EMBASE, and Web of Science up to June 8, 2022. Observational studies and clinical trials on risk stratification in adult and pediatric PAH were included, excluding case reports/series, guidelines, and reviews. Risk of bias was assessed using the Prediction model Risk Of Bias Assessment Tool. Data on the variables used in the models and the predictive strength of the models given by c-statistic were extracted from eligible studies.

**Results:**

A total of 74 studies were eligible for inclusion, with this review focusing on model development (*n* = 21), model validation (*n* = 13), and model enhancement (*n* = 9). The variables used most often in current risk stratification models were the non-invasive WHO functional class, 6-minute walk distance and BNP/NT-proBNP, and the invasive mean right atrial pressure, cardiac index and mixed venous oxygen saturation. C-statistics of current risk stratification models range from 0.56 to 0.83 in adults and from 0.69 to 0.78 in children (only two studies available). Risk stratification models focusing solely on echocardiographic parameters or biomarkers have also been reported.

**Conclusion:**

Studies reporting risk stratification in pediatric PAH are scarce. This systematic review provides an overview of current data on risk stratification models and its value for guiding treatment strategies in PAH.

**Systematic review registration:**

[https://www.crd.york.ac.uk/prospero/display_record.php?ID=CRD42022316885], identifier [CRD42022316885].

## Introduction

Pulmonary hypertension (PH) is a condition defined by an increased pulmonary arterial pressure. Based on pathophysiological mechanisms, clinical presentation, and hemodynamic characteristics, PH can be classified into five main groups: pulmonary arterial hypertension (PAH, group 1), PH due to left heart disease (group 2), PH due to lung disease and/or hypoxia (group 3), PH due to pulmonary artery obstructions (group 4), and PH with unclear and/or multifactorial mechanisms (group 5) (1). Each PH type can be further divided into multiple subgroups. Group 1 PAH is a progressive and eventually fatal pulmonary vascular disease. Occlusion of small pulmonary arteries leads to increased right ventricular afterload, which eventually results in right ventricular failure.

Initially, the only available treatment option for PAH was calcium channel blockers. However, these calcium channel blockers showed only beneficial to a small subset of patients with a response to acute pulmonary vasodilator testing during right heart catheterization (RHC) (2). Over the last decades, various PAH-targeted therapies have become available, such as endothelin receptor antagonists, phosphodiesterase type 5 inhibitors, guanylate cyclase stimulators, prostacyclin analogues, and selective prostacyclin receptor agonists (3). With the availability of these drugs, the treatment of PAH was initially focused on preventing disease progression and prolonging patient survival. When a patient deteriorated on initial therapy, therapy was escalated to double, triple, or maximal combination therapies. These strategies led to improved patient survival after which the focus of treatment strategies started shifting toward clinical improvement. According to current treatment algorithms, treatment decisions are recommended to be based on the assessment of mortality risk of the individual patient, estimated by using clinical prognosticators, both at initiation of therapy as well as for evaluating treatment response (3–5). Therefore, adequate prediction of risk of mortality is pivotal in the treatment of PAH patients.

To estimate patient risk status, various risk equations and risk stratification models have been established. Initially, risk equations were developed to estimate patient outcome by expressing their chances of survival in a percentage. The first time survival was estimated for PAH patients was in 1991 when D’Alonzo et al. (6) developed the NIH (National Institute of Health registry) risk equation, based on the mean pulmonary arterial pressure (mPAP), mean right atrial pressure (mRAP), and cardiac index (CI). Since then other risk equations have been developed, such as the French PAH registry equation (7), the PHC (Pulmonary Hypertension Connection) survival equation (8), and the REVEAL (Registry to Evaluate Early and Long-term Pulmonary Arterial Hypertension Disease Management) risk equation (9). From this original REVEAL risk equation, consisting of nineteen etiologic factors and parameters, the first risk stratification model was derived (10).

Currently, treatment strategies are guided by risk stratification, as proposed by the consecutive European Society of Cardiology/European Respiratory Society (ESC/ERS) guidelines for the diagnosis and treatment of PH (3, 4) and the American College of Cardiology Foundation/American Heart Association (ACCF/AHA) expert consensus document on PH (11). According to these strategies, patients are categorized as having low, intermediate, or high risk for mortality, where the aim is to achieve and maintain a low-risk status. The estimated risk is based on multiple clinical, hemodynamic, and echocardiographic parameters with their own cut-off values for each risk category. A risk stratification guided treatment strategy has also been proposed for children with PAH during the World Symposium on Pulmonary Hypertension (WSPH), using the binary strata low and high risk (12, 13).

The aim of this systematic review is to provide an overview of the current risk stratification models in adult and pediatric PAH. With the growing number of risk stratification models it is crucial to assess the reliability and accuracy of these models, especially since their use in daily practice is advocated. Therefore, the two research questions addressed in this systematic review are: (1) which variables are used for risk stratification models in PAH and (2) what is the prognostic value of risk stratification models for transplant-free survival or all-cause mortality?

## Methods

This review is reported according to the Preferred Reporting Items for Systematic Reviews and Meta-Analysis for Scoping Reviews (PRISMA) (14). The objectives, inclusion criteria and methods adopted in this systematic review were specified and documented in advance (Prospero registration number: CRD42022316885).

### Eligibility criteria

Clinical trials and observational studies focused on risk stratification models both in adult (age ≥ 18 years) and pediatric (age < 18 years) PAH patients were eligible for inclusion. Pediatric patients with PH due to lung disease were also considered eligible for inclusion because of the pathological crossover between PAH and the abnormal pulmonary vascular development, seen in developmental lung diseases such as bronchopulmonary dysplasia and congenital diaphragmatic hernia. In these studies the diagnosis had to be confirmed by RHC, or echocardiography in infants with developmental lung disease, and meet the hemodynamic definitions (1). Additionally, the risk stratification model was considered a model only if it comprised at least three variables. Results and conclusions had to be supported by appropriate statistical methods with endpoints defined as transplant-free survival or all-cause mortality. Furthermore, studies had to be written in English.

Studies reporting risk stratification models in adult patients with PH group 2, 3, 4, and 5 according to the Nice 2018 classification (1), and pediatric patients with PH group 2, 4, and 5 were excluded, as well as case reports, case series, guidelines, and reviews. If less than three variables were used for risk stratification models or endpoints other than transplant-free survival or all-cause mortality, studies were excluded as “no risk stratification model” or “not eligible endpoint,” respectively. Studies not meeting the inclusion criteria and not fitting any of the above mentioned exclusion reasons were excluded as “other.” In this review, survival or risk equations were not considered as risk stratification models.

### Information sources and search strategy

Systematic literature searches were conducted in the electronic databases MEDLINE (PubMed), Embase (Elsevier), and Web of Science (Clarivate). The search strategies were developed in collaboration with an information specialist (SW). The structure of the search strategies is based on two concepts: (1) PAH, pediatric PH and (2) risk stratification, risk tooling, prediction modeling. For each concept a search block was developed based on index terms and free text words including synonyms and related terms. No time or language restrictions were applied. The search strategies were initially run at March 3, 2022 and updated at June 8, 2022. The full search strategies can be found in Supplementary Table 1.

### Managing references and selection process

The results of the database searches were exported to the reference management program EndNote, version 20. In EndNote, duplicate items were determined and removed following the steps described by Bramer et al. (15). The de-duplicated results were exported to the screening program Rayyan.

Two researchers independently performed the screening in Rayyan in two steps. In the title-abstract screening, articles were excluded that were clearly not relevant. Potentially relevant articles and articles with insufficient information in the titles or abstract selected by at least one of the researchers were selected for the full-text screening. In the full-text screening, the two researchers independently judged if the selection criteria were met. Disagreements in decisions between the screeners were solved by a third reviewer. Finally, articles that met the criteria, as agreed by the researchers, were included and divided into four classes judged on the primary aim of the article: (1) model development, (2) model validation, (3) model enhancement, and (4) serial risk stratification. In accordance with the aims of the systematic review, the authors focused on the studies belonging to class 1, 2, and 3. Studies in class 4 focused on risk stratification at follow-up and/or changes in risk score or stratum between baseline and follow-up, whether or not under the influence of intervention, and were hence disregarded from the current review.

### Data collection

Data was extracted from the included studies using a standardized data extraction form. Extracted data included: study setting, population demographics and baseline characteristics, variables used in the risk stratification model including cut-off points and defined endpoint, statistical methodology, and the prognostic value of the model.

### Analysis

To present an overview of the variables used in risk stratification models, multiple tables were produced. Each table reports the model name or basis, the used definition of risk, the number of risk strata, the number of variables, and specifies which variables are used for the model. Separate tables were created for the renowned risk stratification models (containing both development and validation), the lesser studied models, model enhancement, and pediatric risk stratification strategies.

For the evaluation of the prognostic value of the risk stratification models, the reported c-statistic was used. The c-statistic is equivalent to the area under the receiver operating characteristic curve (AUROC) and is a measure of the discriminatory ability. It can be interpreted as the probability that a patient who died had a higher predicted probability of death than a patient who survived. A c-statistic of 1.0 shows a perfect prediction, whereas a c-statistic of 0.5 is indicative of poor prediction and the model is no better than chance. Hence, the model with the higher c-statistic (or greater AUROC) is better at discriminating between survival and death (17).

### Risk of bias

Risk of bias (ROB) was assessed using the Prediction model Risk Of Bias Assessment Tool (PROBAST) for every study and in case of studies including multiple models, separately for the different risk stratification models (16). This tool consists of four domains - participants, predictors, outcome, and analysis–with a total of 20 signaling questions to assist in assessing ROB. These questions can be answered as (probably) yes, (probably) no, or no information, with “no” indicating potential bias. For model development studies, the development signaling questions were answered, and for validation studies the validation questions. In the case of studies reporting both the development of a model and the validation of this model or other models, both the development and validation signaling questions were answered for each model separately. For model enhancement studies, the development questions were acknowledged, as well as the validation questions when the original model was also validated. Besides ROB, the applicability of the model was evaluated to determine the relevance of the participants, predictors, and outcome to the research question. ROB and applicability assessment was performed by one researcher, but when in doubt, a second researcher was consulted.

## Results

### Identified studies

**FIGURE 1:**
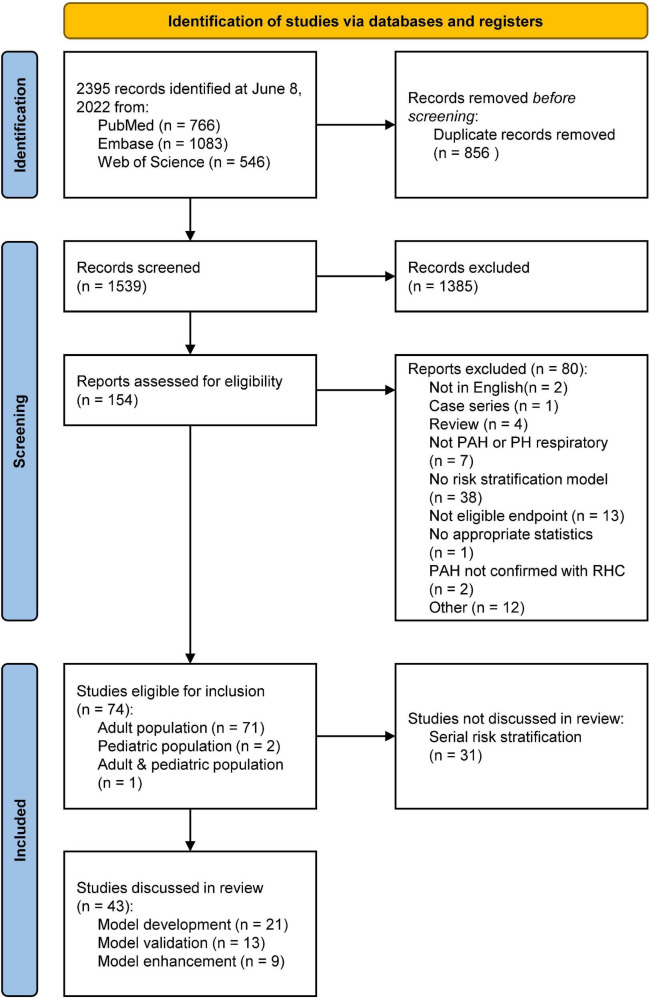
PRISMA flowchart showing the study selection. PAH, pulmonary arterial hypertension; PH, pulmonary hypertension; RHC, right heart catheterization. Other reasons included editorials, retracted articles, and commentaries.

In Figure 1, the PRISMA flowchart for the identification of studies is shown. A total of 2,395 records were identified from the databases. After duplicate removal, 1,539 studies remained for abstract screening of which 1,385 were excluded during abstract screening. Of the 154 full-text screened studies, 80 were excluded (Supplementary Table 2). The remaining 74 studies were considered eligible for inclusion (Supplementary Table 3), of which two studies involved pediatric PAH patients. No studies concerning risk stratification in pediatric PH due to lung disease were retrieved, as such our results focus on RHC confirmed PAH only. The 31 studies concerning serial risk stratification were disregarded, since the current study focusses solely on model development (*n* = 21), validation (*n* = 13), and enhancement (*n* = 9) of risk stratification models, resulting in a total of 43 studies to be discussed in this review. The main characteristics of these 43 studies are presented in Tables 1–3 for respective development, validation, and enhancement studies.

**TABLE 1:** Study characteristics of studies describing model development.

Study	Cohort	Study site	Study baseline	Endpoint	C-statistic	Patients *n*	Age (years)	Female%	IPAH/HPAH%	PAH-CHD%	PAH-CTD%	PAH other%	WHO-FC	6MWD (m)	NT-proBNP (pg/ml)	mRAP (mmHg)	mPAP (mmHg)	CI (L/min/m^2^)	PVR (WU)
Austin (37)	Der	USA Florida	E	TFS	1 year	175	60	69	43	5	35	17	2.7	299	358^	8.7	45	2.4	8.3*
	Val	USA Rochester	E	TFS	1 year	677	53.7	75	48	11	28	13		327			52	2.5	
Benza (10)	Der	USA multicenter	E	ACM	1 year	2716	50.4	78.6	49.4	11.8	23.9	15	2.5	370	1455	8.6	49.5	2.6	10.5*
	Val	USA multicenter	D	ACM	1 year	504	53	74	50	4.8	31	14.3	2.9	308	2705	10.0	48.8		10.7
Benza (18)		USA multicenter	1 year post E	ACM	1 year	2529	53.6	80	48.2	9.6	25.7	16.5	2.5	374	1730	8.7	48.5		9.6
Benza (19)		USA multicenter	1 year post E	ACM	1 year	2529	53.6	80	48.2	9.6	25.7	16.5	2.5	374	1730	8.7	48.5		9.6
Boucly (23)		Canada Ottawa	D	TFS	5 year	211	63.2	64.6	69.8	6.6	23.7		2.8	272	3152	7.9	44.9	2.17	9.8
Chiu (24)		Taiwan multicenter	E	ACM		87	37.4	31		100			2.4	385	1748	6.5	56.7	2.4	11.2
Dardi (25)		Italy Bologna	D	ACM	1 year	725	51*	69	55.9	20.2	23.9		2.9	389*	807*	7*	53*	2.4*	11*
Ghio (38)		EU and USA multicenter	E	ACM		517	52	61.1	64.8	8.4	14.1	12.7	2.8	358					
Haarman (45)		Netherlands Groningen	D	TFS	5 year	58	6.8*	53.4	100				2.8						
Haddad (41)		USA Stanford	E	TFS	5 year	231	48*	78.8			32		2.6	430*	407*	7.0*	50.1	2.0*	
Hoeper (20)		EU multicenter	D	ACM		1588	64	64	67	4	22	7	3.0	298	1573*	8	45	2.3	9.8
Hoeper (22)		EU multicenter	D	ACM		1655	65.7	64.3	71.4	2.8	19.9	5.8	3.0	293	1499*	8.2	43.3	2.2	9.3
Imai (26)		Japan Nagoya	E	ACM		80	48	79	36	13	36	15	2.5	370	75*^	5.9	45	2.9	8.9
Kylhammar (21)		Sweden multicenter	D	ACM		530	68**	64.7	50.6	12.6	30.6	6.2							
Lee (52)	Der	UK Glasgow	D	ACM		182	62*	69	54		32	13	2.9	260*	1026*	7*	47*		10.8*
	Val	UK Cambridge	D	ACM		99	53*	27	100				2.9	267*	2029*	9*	50*		13.3*
Li (27)		China Nanjing	D	ACM		50	39.1	94			100		2.8	370	1790	7	45.6	2.7	10.6
Mercurio (28)		USA Baltimore	D	ACM		151	61	84.8			100^††^								
Rhodes (39)	Der**	UK multicenter	E	TFS	5 year	238	39.1*	74	100				2.9	353*		9*	55*	2.0*	12.6*
	Val	France multicenter	D	TFS	5 year	79	41.9*		84.8			15.2	2.6	360*		8*	50*	2.39*	9.9*
Wang (29)		China multicenter	D	ACM		103	43.2	98			100^†^		2.3	398	822*	6.4	48.1	2.6	11.5
Xiong (31)	Der	China Shanghai	E	ACM	1 year	108	52.8	71.3	47.2	9.3	32.4	11.1	2.7	338	3268	9.7	45.2		10.5
	Val	China Shanghai	E	ACM	1 year	216	54.6	73.6	49.1	11.6	28.7	14.6	2.7	309	3497	10.6	46.7		11.2
Yogeswaran (40)	Der	Germany Giessen	D	ACM		227	49*	67	100				3.0	335	265^		50.5	2.1	11.2*
	Val	Germany Hamburg	D	ACM		234	67*	64						309	2960		44.0	2.3	7.8*

Values reported as mean, unless stated otherwise. PAH, pulmonary arterial hypertension; IPAH, idiopathic PAH; HPAH, hereditary PAH; CHD, congenital heart disease; CTD, connective tissue disease; WHO-FC, WHO functional class; 6MWD, 6-minute walk distance; NT-proBNP, N-terminal-pro brain natriuretic peptide; mRAP, mean right atrial pressure; mPAP, mean pulmonary arterial pressure; CI, cardiac index; PVR, pulmonary vascular resistance; Der, derivation; Val, validation; D, diagnosis; E, enrollment; TFS, transplant-free survival; ACM, all-cause mortality. *Median, **subgroup, ^$^indexed PVRI, ^BNP, ^†^primary Sjögren’s syndrome, and ^††^systemic sclerosis.

**TABLE 2:** Study characteristics of studies describing model validation.

Study	Cohort	Study site	Study baseline	Endpoint	C-statistic	Patients *n*	Age (years)	Female%	IPAH/HPAH%	PAH-CHD%	PAH-CTD%	PAH other%	WHO-FC	6MWD (m)	NT-proBNP (pg/ml)	mRAP (mmHg)	mPAP (mmHg)	CI (L/min/m^2^)	PVR (WU)
Anderson (53)		Australia and New Zealand multicenter	1 year post E	ACM	1 year	1011	58.2	77.1	49	9.9	31.0	10.1	2.6	383.7		8.5			7.5
Boucly (42)		France multicenter	D	ACM	1 year	2879	61	60	43	1	27	29	2.7	300*	995*	8	45	2.6	8.8
Chang (54)		USA multicenter	E	ACM		935	56*	76	43	5	32	14	2.6	335	603*	10	49.5	2.3	9.1*
Gong (55)		China Shanghai	D	ACM		392	40	67	100				2.6	379	748*	6*	58*	2.4*	14*
Hjalmarsson (56)		Sweden multicenter	D	TFS		502	68*	65	61		39		2.9	267*	1573*	7*	45*	2.3*	9*
Kylhammar (57)		Sweden multicenter	D	TFS		252	53*	73	46	13	33	8	2.8	373*	803*	6*	47*	2.5*	8.7*
Mullin (58)	JHPHP	USA Baltimore	D	ACM	1 year	117	62.3	81.2			100^††^		2.6	319*	942*	8	40	2.5	8.1
	PHAROS	USA and Canada multicenter	E	ACM	1 year	175	60	88.9			100^††^		2.4	366*	331.5*		37		6.5
Qu (59)		China multicenter	D	ACM	1 year	306	35	99.3			100^¥^		2.5	409	1848	5.9	46.9		11.0
Quan (46)		China multicenter	D	ACM	1 year	2031	35	76.2	38.8	45.2	13.1	3.0	2.2	412	1393	6.5	59.8	3.1	13.7
Sitbon (60)		France multicenter	E	ACM	1 year	1737	54.7	58.8	41.2	8.7	21.5	28.6	2.7	356		7.7	48.3	2.6	9.7
Vraka (61)		Switzerland Lausanne	D	TFS	3 year	50	54.8	68	56	8	14	22	2.8	326	1847	8.6	47.4	2.6	9.6
Weatherald (48)		France multicenter	D	TFS	1 year	513	67.8*	78.3			100		2.8	285*	1144*	6*	40*	2.5*	7.5*
Xanthouli (30)		Germany Heidelberg	D	ACM		142	63.3	61.3	33.8		26.1		2.9	333	2334	7.9	43.2	2.4	8.1

Values reported as mean, unless stated otherwise. PAH, pulmonary arterial hypertension; IPAH, idiopathic PAH; HPAH, hereditary PAH; CHD, congenital heart disease; CTD, connective tissue disease; WHO-FC, WHO functional class; 6MWD, 6-minute walk distance; NT-proBNP, N-terminal-pro brain natriuretic peptide; mRAP, mean right atrial pressure; mPAP, mean pulmonary arterial pressure; CI, cardiac index; PVR, pulmonary vascular resistance; D, diagnosis; E, enrollment; TFS, transplant-free survival; ACM, all-cause mortality; JHPHP, Johns Hopkins Pulmonary Hypertension Program; PHAROS, Pulmonary Hypertension Assessment and Recognition of Outcome in Scleroderma Registries. *Median, ^††^systemic sclerosis, and ^¥^systemic lupus erythematosus.

**TABLE 3:** Study characteristics of studies describing model enhancement.

Study	Cohort	Study site	Study baseline	Endpoint	C-statistic	Patients *n*	Age (years)	Female%	IPAH/HPAH%	PAH-CHD%	PAH-CTD%	PAH other%	WHO-FC	6MWD (m)	NT-proBNP (pg/ml)	mRAP (mmHg)	mPAP (mmHg)	CI (L/min/m^2^)	PVR (WU)
Griffiths (44)		USA multicenter	E	ACM		182	13*	59	52.2	37.9	3.3	6.6	2.4	423*	218*	7*	54*	3.7*	10.7*^$^
Harbaum (43)		Germany multicenter	D	TFS		204	56	67	96			4	2.8	340	2932	9	59	2.2	12
Kanwar (36)	Internal validation REVEAL 2.0	USA multicenter	E	ACM	1 year		53.6	80.0	49.2		25.7	13.4	2.5						
	External validation COMPERA	EU multicenter	D	ACM	1 year			64.3	48.3		35.0	7	2.9						
	External validation PHSANZ	Australia and New Zealand multicenter	E	ACM	1 year			77.7	49.9		32.2	17.4	2.6						
Lewis (34)		UK multicenter	D	ACM	1 year	438	56.6	75	45	9	37	9	2.8			10	48	2.8	8.9
Lewis (62)		UK multicenter	D	TFS	1 year	1240	64*	71	48.6		51.4		3.1			9*	48*	2.4*	9.1*
Simpson (35)		USA multicenter	E	ACM		2017	55	80	43.1		30.9	26	2.6	347	672*	9	50	2.7	10
Vicenzi (63)		Belgium Brussels	D	TFS		102	54	62.7	57.8	13.7	14.7	13.8	3.0	415*	1077*				
Yogeswaran (32)		Germany Giessen	D	ACM		301	58	65								6.3	45		8.9
Zelt (33)		Canada Ottowa	D	TFS	5 year	211	63.2	64.6	69.8	6.6	23.7		2.8	272	3152	7.9	44.9	2.17	9.8

Values reported as mean, unless stated otherwise. PAH, pulmonary arterial hypertension; IPAH, idiopathic PAH; HPAH, hereditary PAH; CHD, congenital heart disease; CTD, connective tissue disease; WHO-FC, WHO functional class; 6MWD, 6-minute walk distance; NT-proBNP, N-terminal-pro brain natriuretic peptide; mRAP, mean right atrial pressure; mPAP, mean pulmonary arterial pressure; CI, cardiac index; PVR, pulmonary vascular resistance: D, diagnosis; E, enrollment; TFS, transplant-free survival; ACM, all-cause mortality. *Median and ^$^PVRI.

### Variables in risk stratification

We have identified multiple risk stratification models, such as the REVEAL risk calculator and the ESC/ERS 2015 guidelines-based COMPERA, SPARH, FPRH invasive and non-invasive models, and other abbreviated versions of the ESC/ERS 2015 guidelines. In Table 4 an overview of the variables used for these risk stratification models is given, along with the total number of variables used in each model, the risk definition, and the number of strata.

**TABLE 4:** Variables used in REVEAL and ESC/ERS 2015 guideline-based risk stratification models: development and validation studies.

Study	Model name	Definition of risk	Strata	Number of variables	WHO-FC	6MWD	NT-proBNP	BNP	Peak VO_2_	RA area	Pericardial effusion	mRAP	CI	SvO_2_	PVR	WHO group 1 subgroup	Male age > 60	Renal insufficiency	eGFR	Systolic BP	Heart Rate	% predicted DL_*CO*_	All-cause hospitalizations ≤ 6 months	Syncope	Clinical signs RHF
Benza (10), Austin (37), Mullin (58), Qu (59), Sitbon (60), Xiong (31), Zelt (33)	REVEAL risk score calculator	Risk category based on total score	5	12																					
Benza (18), Anderson (53), Harbaum (43), Lewis (34), Quan (46), Simpson (35), Vraka (61), Zelt (33)	REVEAL 2.0 calculator	Risk category based on total score	3	13			OR	OR										OR	OR						
Benza (19), Chang (54), Quan (46)	REVEAL Lite 2	Risk category based on total score	3	6			OR	OR										OR	OR						
Hoeper (20), Benza (18), Chang (54), Dardi (25), Gong (55), Harbaum (43), Quan (46), Vicenzi (63), Vraka (61), Xanthouli (30), Yogeswaran (40)	COMPERA model	Risk category based on average score	3	6			OR	OR																	
Hoeper (22), Boucly (42)	COMPERA model (abbreviated)	Risk category based on average score	3	3			OR	OR																	
Hoeper (22), Boucly (42)	COMPERA 2.0 model	Risk category based on average score	4	3			OR	OR																	
Kylhammar (21), Hjalmarsson (56), Kylhammar (57)	SPAHR model	Risk category based on average score	3	8																					
Boucly (23), Benza (18), Chang (54), Dardi (25), Mercurio (28), Quan (46), Simpson (35), Vicenzi (63), Vraka (61), Weatherald (48), Zelt (33)	FPHR invasive model	#low risk criteria	3	4																					
Boucly (23), Harbaum (43), Quan (46), Simpson (35), Vicenzi (63), Xanthouli (30)	FPHR non-invasive model	#low risk criteria	3	3			OR	OR																	
Chiu (24)	ESC/ERS 2015 (abbreviated, non-invasive)	#high risk criteria	2	3																					
Dardi (25)	ESC/ERS 2015 (abbreviated)	Other	3	6			OR	OR																	
Imai (26)	ESC/ERS 2015 (abbreviated)	Risk category based on average score	3	7																					
Li (27)	ESC/ERS 2015 (abbreviated)	Other	3	7																					
Mercurio (28)	ESC/ERS 2015 (abbreviated)	Risk category based on average score	3	5																					
Wang (29)	ESC/ERS 2015 (abbreviated)	Other	3	6			OR	OR																	
Xanthouli (30)	ESC/ERS 2015 (abbreviated, non-invasive)	Risk category based on average score	3	4																					
Xiong (31)	ESC/ERS 2015 (abbreviated, non-invasive)	Risk category based on total score	3	4																					
Yogeswaran (32)	ESC/ERS 2015 (abbreviated)	Risk category based on average score	3	5																					
Zelt (33)	ESC/ERS 2015 (abbreviated)	Risk category based on average score	3	10																					

WHO-FC, WHO functional class; 6MWD, 6-minute walk distance; NT-proBNP, N-terminal-pro brain natriuretic peptide; BNP, brain natriuretic peptide; peak VO_2_, peak oxygen consumption; RA area, right atrial area; mRAP, mean right atrial pressure; CI, cardiac index; SvO_2_, mixed venous oxygen saturation; PVR, pulmonary vascular resistance; eGFR, estimated glomerular filtration rate; BP, blood pressure; DL_CO_, carbon monoxide lung diffusing capacity; RHF, right heart failure.

The first REVEAL risk calculator was developed by Benza et al. (10) in 2012 and consisted of twelve variables: WHO Functional Class (WHO-FC), 6-minute walk distance (6MWD), N-terminal-pro brain natriuretic peptide (NT-proBNP, or brain natriuretic peptide–BNP), pericardial effusion, mRAP, pulmonary vascular resistance (PVR), WHO group 1 subgroup, male older than 60 years of age, renal insufficiency, systolic blood pressure (SBP), heart rate (HR), and percentage predicted carbon monoxide lung diffusing capacity (DL_*CO*_). Points are assigned to every variable, with its weight based on the results of the multivariable Cox proportional hazard model. In 2019, Benza et al. (18) updated some of the cut-off values of the variables and added an extra variable to the model, all-cause hospitalizations within the last 6 months, resulting in the REVEAL 2.0 calculator. The REVEAL Lite 2, a non-invasive, abbreviated version of the REVEAL 2.0 calculator, was published by Benza et al. (19) in 2021.

Many different methods have been developed based on the risk stratification as recommended by the ESC/ERS 2015 guidelines. In 2017, Hoeper et al. (20) reported the COMPERA (Comparative, Prospective Registry of Newly Initiated Therapies for Pulmonary Hypertension) model which uses six variables: WHO-FC, 6MWD, NT-proBNP (or BNP), mRAP, CI, and mixed venous oxygen saturation (SvO_2_). Each variable is assigned a grade 1 (low risk), 2 (intermediate risk), or 3 (high risk) according to the cut-off values derived from the ESC/ERS 2015 guidelines. To determine the risk class, the sum of these grades is divided by the number of available variables and rounded to the nearest integer. Kylhammar et al. (21) created a similar method with SPAHR (Swedish PAH Register), but included two more variables: right atrial (RA) area and pericardial effusion. Since many patients were stratified as having intermediate risk, Hoeper et al. (22) created the abbreviated version COMPERA 2.0 model in 2021, where the intermediate risk stratum is split into intermediate-low and intermediate-high risk, resulting in a four-strata model consisting of three variables: WHO-FC, 6MWD, and NT-proBNP. The FPHR (French pulmonary hypertension registry) invasive and non-invasive method, published by Boucly et al. (23) in 2017, uses the number of low-risk criteria to estimate the mortality risk. WHO-FC, 6MWD, mRAP, and CI are used in the invasive method, whereas WHO-FC, 6MWD, and NT-proBNP (or BNP) are used in the non-invasive method. A major limitation of this method is that it cannot be applied if one of the variables is missing. Besides COMPERA, SPAHR, and FPHR models, other abbreviated versions of the ESC/ERS 2015 guidelines were reported (24–33). The variables used in these models are also shown in Table 4. From this table it can be observed that most often used variables in risk stratification models are WHO-FC, 6MWD, NT-proBNP, mRAP, CI, and SvO_2_.

The enhancement of above mentioned risk stratification models has been explored by several studies by adding one or more imaging or biomarker variables to the models, such as the right ventricular end-systolic volume index (34), estimated glomerular filtration rate (eGFR) (33), or endostatin (35). To enhance the performance of the REVEAL 2.0 calculator, Kanwar et al. (36) produced a tree-augmented naïve Bayes version using the same variables and cut-off values as the REVEAL 2.0 calculator. In Table 5 an overview of the model enhancement studies with the variables is presented.

**TABLE 5:** Variables used in REVEAL and ESC/ERS 2015 guideline-based risk stratification models: enhancement studies.

Study	Model name	Definition of risk	Strata	Number of variables	WHO-FC	6MWD	NT-proBNP	BNP	Pericardial effusion	mRAP	CI	SvO_2_	PVR	WHO group 1 subgroup	Male age > 60	Renal insufficiency	eGFR	Systolic BP	Heart Rate	% predicted DL_*CO*_	All-cause hospitalizations ≤ 6 months	Syncope	Clinical signs RHF	TAPSE/sPAP	PaCO_2_	ISWD	RVESVi	TAPSE/TRV	Endostatin
Zelt (33)	REVEAL risk score calculator + eGFR	Risk category based on total score	3	12												OR	OR												
Harbaum (43)	REVEAL 2.0 calculator + PaCO_2_	Risk category based on total score	3	14			OR	OR								OR	OR												
Lewis (34)	REVEAL 2.0 calculator + RVESVi	Risk category based on total score	3	14			OR	OR								OR	OR												
Lewis (62)	REVEAL 2.0 calculator ISWD	Risk category based on total score	3	13			OR	OR								OR	OR												
Simpson (35)	REVEAL 2.0 calculator + endostatin	Risk category based on total score	3	14			OR	OR								OR	OR												
Kanwar (36)	REVEAL 2.0 tree-augmented naïve Bayes	Other	3	13			OR	OR								OR	OR												
Harbaum (43)	COMPERA model + PaCO_2_	Risk category based on average score	3	7			OR	OR																					
Vicenzi (63)	COMPERA model + TAPSE/TRV or TAPSE/sPAP	Risk category based on average score	4	7			OR	OR																OR				OR	
Lewis (34), Lewis (62)	FPHR invasive model ISWD	#low risk criteria	3	4																									
Lewis (34)	FPHR invasive model ISWD + RVESVi	#low risk criteria	3	5																									
Simpson (35)	FPHR invasive model + NTproBNP	#low risk criteria	3	5																									
Simpson (35)	FPHR invasive model + endostatin	#low risk criteria	3	5																									
Simpson (35)	FPHR invasive model + NTproBNP + endostatin	#low risk criteria	3	6																									
Vicenzi (63)	FPHR invasive model + TAPSE/TRV or TAPSE/sPAP	#low risk criteria	4	5																				OR				OR	
Zelt (33)	FPHR invasive model + eGFR	#low risk criteria	3	5																									
Harbaum (43)	FPHR non-invasive model + PaCO_2_	#low risk criteria	3	4			OR	OR																					
Simpson (35)	FPHR non-invasive model + endostatin	#low risk criteria	3	4			OR	OR																					
Vicenzi (63)	FPHR non-invasive model + TAPSE/TRV or TAPSE/sPAP	#low risk criteria	4	4			OR	OR																OR				OR	
Yogeswaran (32)	ESC/ERS 2015 (abbreviated) + TAPSE/sPAP	Other	4	6																									
Zelt (33)	ESC/ERS 2015 (abbreviated) + eGFR	Risk category based on average score	3	11																									

WHO-FC, WHO functional class; 6MWD, 6-minute walk distance; NT-proBNP, N-terminal-pro brain natriuretic peptide; BNP, brain natriuretic peptide; mRAP, mean right atrial pressure; CI, cardiac index; SvO_2_, mixed venous oxygen saturation; PVR, pulmonary vascular resistance; eGFR, estimated glomerular filtration rate; BP, blood pressure; DL_CO_, carbon monoxide lung diffusing capacity; RHF, right heart failure; TAPSE/sPAP, tricuspid annular plane systolic excursion/systolic pulmonary artery pressure ratio; PaCO_2_, arterial carbon dioxide partial pressure; ISWD, incremental shuttle walk distance; RVESVi, right ventricular end-systolic volume index; TAPSE/TRV, tricuspid annular plane systolic excursion/tricuspid regurgitation velocity ratio.

Additionally, others have tried to create risk stratification models based solely on echocardiographic parameters (37, 38) or biomarkers (39, 40) (Table 6). For example, Ghio et al. (38) used the echocardiographic parameters tricuspid annular plane systolic excursion (TAPSE), degree of tricuspid regurgitation (TR) and a marker of systemic venous congestion represented by inferior vena cava diameter. Yogeswaran et al. (40) developed a model with the biomarkers ƴ-glutamyl transferase (GGT), aspartate aminotransferase/alanine aminotransferase (AST/ALT) ratio, and neutrophil-to-lymphocyte ratio (NLR). A different approach for developing a risk stratification model was shown by Haddad et al. (41). They attempted to model the data architecture by creating a network graph. This graph shows the connectivity of every parameter with the other parameters and identified NT-proBNP as the most central (important) parameter.

**TABLE 6:** Variables used in other risk stratification models.

Study	Model name	Definition of risk	Strata	Number of variables	6MWD	NT-proBNP	Pericardial effusion	mRAP	TAPSE	WHO group 1 subgroup	Systolic BP	Heart Rate	% predicted DL_*CO*_	Age	Sex	CO	CTD etiology	TR	dilated IVC	eRAP	RVESRI	Sodium	Albumin	GGT	AST/ALT	NLR	SVEP1	PXDN	Renin	NRP1	TSP2	PRDX4
Austin (37)	Echocardiographic approach	# high risk criteria	2	3																												
Ghio (38)	Echocardiographic approach	low risk: TAPSE > 17 mm and TR grade 0–1; intermediate risk: TAPSE > 17 mm and TR 2–3 OR TAPSE ≤ 17 mm and normal IVC; high risk: TAPSE ≤ 17 mm and dilated IVC	3	3																												
Haddad (41)	Eigenvector centrality model	based on the normally distributed prognostic index (weighted sum of coefficients)	5	9																												
Haddad (41)	Eigenvector centrality model–biomarker-focused	based on the normally distributed prognostic index (weighted sum of coefficients)	5	7																												
Haddad (41)	Eigenvector centrality model – imaging-focused	Based on the normally distributed prognostic index (weighted sum of coefficients)	5	8																												
Lee (52)	Scottish composite score	Risk category based on total score	3	6																												
Rhodes (39)	Plasma proteome 6 + NT-proBNP	Risk category based on total score	2	7																												
Rhodes (39)	Plasma proteome 6	Risk category based on total score	2	6																												
Yogeswaran (40)	Biomarker approach	Risk category based on average score	3	3																												

6MWD, 6-minute walk distance; NT-proBNP, N-terminal-pro brain natriuretic peptide; mRAP, mean right atrial pressure; TAPSE, tricuspid annular plane systolic excursion; BP, blood pressure; DL_CO_, carbon monoxide lung diffusing capacity; CO, cardiac output; CTD, connective tissue disease; TR, tricuspid regurgitation; IVC, inferior vena cava; eRAP, estimated right atrial pressure; RVESRI, right ventricular end-systolic volume index; GGT, ƴ-glutamyl transferase; AST/ALT, aspartate aminotransferase/alanine aminotransferase ratio; NLR, neutrophil-to-lymphocyte ratio.

### Prognostic value of risk stratification models

**FIGURE 2:**
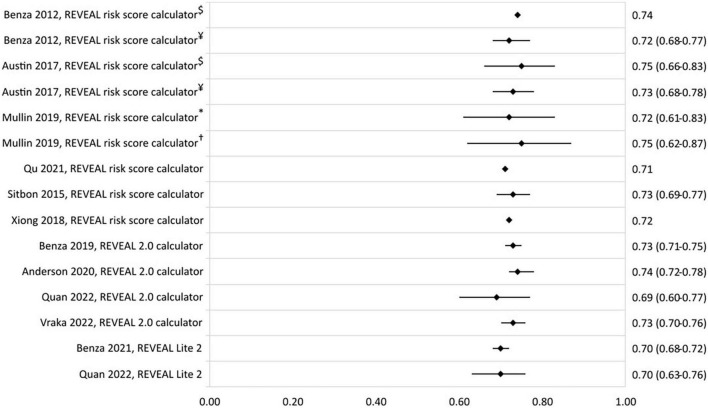
C-statistic (95% CI) of the development and validation of REVEAL risk stratification models. ^$^Derivation cohort, ^¥^validation cohort, *JHPHP, and ^†^PHAROS.

The prognostic value of the REVEAL risk scores in different studies and populations is shown in Figure 2 by a forest plot of the c-statistic with its 95% confidence interval (95% CI). The c-statistic was found to range from 0.70 to 0.75 for the REVEAL risk score calculator and from 0.65 to 0.74 for the REVEAL 2.0 calculator. REVEAL Lite 2 had a c-statistic of 0.70.

**FIGURE 3:**
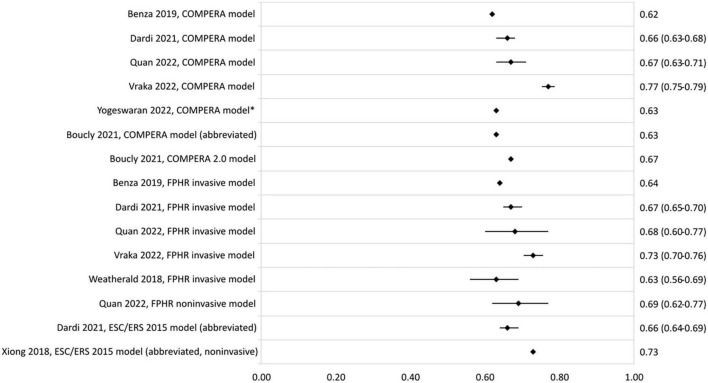
C-statistic (95% CI) of the development and validation of ESC/ERS 2015 guideline-based models. *Cohort includes PAH and chronic thromboembolic PH patients.

In Figure 3, the c-statistics of the risk stratification models based on the ESC/ERS 2015 guidelines are presented. C-statistic ranged from 0.62 to 0.77 for the COMPERA model, and from 0.56 to 0.73 and 0.39 to 0.69 for the FPHR invasive and non-invasive method, respectively. The COMPERA 2.0 model showed a c-statistic of 0.67 in a validation study by Boucly et al. (42). Other abbreviated versions of the ESC/ERS 2015 guidelines c-statistic ranged from 0.60 to 0.73. Highest c-statistic was reported by Xiong et al. (31) with a model consisting of the non-invasive variables WHO-FC, 6MWD, NT-proBNP, and RA area.

**FIGURE 4:**
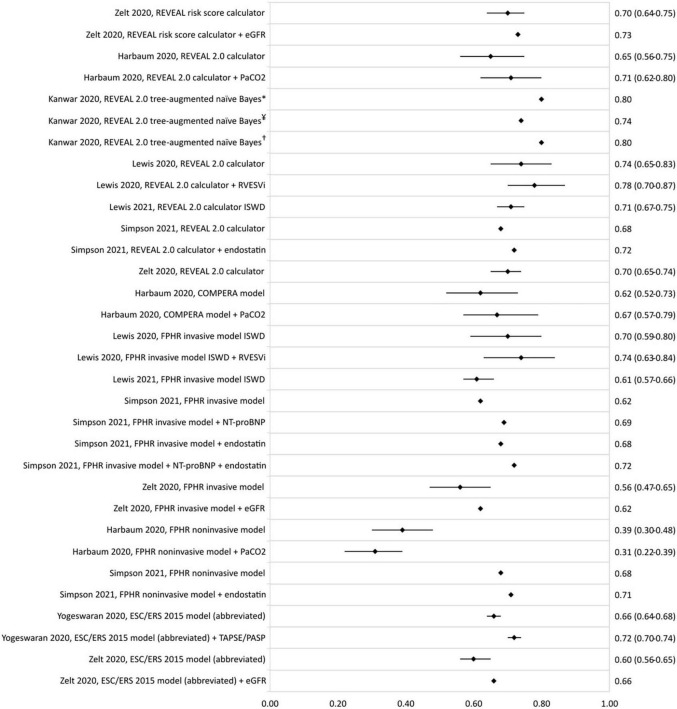
C-statistic (95% CI) of the enhancement of REVEAL and ESC/ERS 2015 guideline-based risk stratification models, as well as the c-statistic of the original model in the same population. eGFR, estimated glomerular filtration rate; PaCO2, arterial carbon dioxide partial pressure; RVESVi, right ventricular end-systolic volume index; ISWD, incremental shuttle walk distance; NT-proBNP, N-terminal-pro brain natriuretic peptide; TAPSE/PASP, tricuspid annular plane systolic excursion/systolic pulmonary artery pressure ratio. *REVEAL cohort, ^¥^COMPERA cohort, and ^†^PHSANZ cohort.

Several enhancement studies were found to have an increase in c-statistic upon the addition of an imaging or serum biomarker to a previously described model (Figure 4). Lewis et al. reported an increase in c-statistic of the REVEAL 2.0 calculator from 0.74 (0.65–0.83) to 0.78 (0.70–0.87) upon addition of the right ventricular end-systolic volume index. Harbaum et al. (43) increased the c-statistic of the COMPERA model from 0.62 (0.52–0.73) to 0.67 (0.57–0.79) by adding arterial carbon dioxide partial pressure to the model. Addition of biomarkers NT-proBNP and endostatin to the FPHR invasive method was shown to increase the c-statistic from 0.62 to 0.72 (35), and endostatin also increased the c-statistic of the FPHR non-invasive method from 0.68 to 0.71 (35).

**FIGURE 5:**
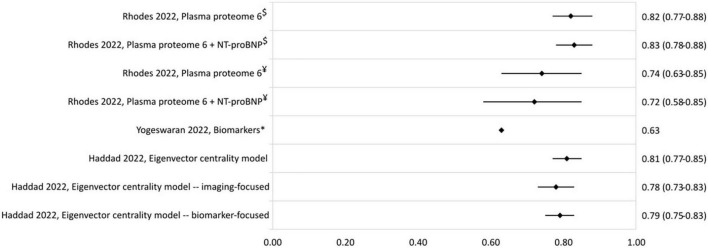
C-statistic (95% CI) of biomarker and eigenvector centrality risk stratification models. NT-proBNP, N-terminal-pro brain natriuretic peptide. ^$^Derivation cohort, ^¥^validation cohort, *cohort includes PAH and chronic thromboembolic PH patients.

The c-statistics of other developed risk stratification models are presented in Figure 5. A model using the plasma proteome had c-statistics of 0.82 (0.77–0.88) and 0.74 (0.63–0.85) in the derivation and validation cohort, respectively (39). The eigenvector centrality model developed by Haddad et al. (41) had a c-statistic of 0.81 (0.77–0.85).

### Risk stratification in pediatric pulmonary arterial hypertension

**FIGURE 6:**
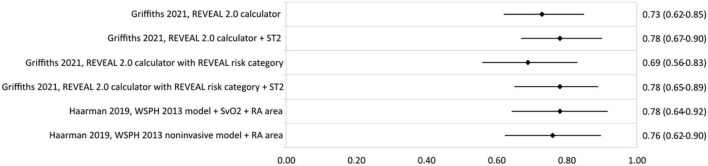
C-statistic (95% CI) of risk stratification models used in pediatric PAH. ST2, soluble suppressor of tumorigenicity-2; SvO2, mixed venous oxygen saturation; RA area, right atrial area.

Only two studies reporting risk stratification in pediatric PAH were found eligible for inclusion. Griffiths et al. (44) applied the REVEAL 2.0 calculator in 182 children with a median age (interquartile range–IQR) of 13 (8–17) years. They used the variables and cut-off values from the REVEAL 2.0 calculator, except for renal insufficiency, and categorized the patients according to the five risk strata from the original REVEAL risk score calculator (Table 7). The reported c-statistic (Figure 6) of the model in this pediatric population was 0.69 (0.56–0.83). Addition of soluble suppressor of tumorigenicity-2 increased the c-statistic to 0.78 (0.65–0.89). The other pediatric PAH study was by Haarman et al. (45) and described the development of two risk stratification models in 58 children with a median age of 6.8 (2.2–13.4) years. The models were based on the variables and cut-off values recommended by the WSPH 2013 pediatric task force (12) with the addition of two variables from the ESC/ERS 2015 guideline (3) and risk was defined as the number of low risk criteria. The first model consisted of the following variables: WHO-FC, NT-proBNP, mRAP, CI, TAPSE, syncope, height, body mass index, mPAP/mean systemic arterial pressure ratio, indexed PVR, acute vasoreactivity, SvO_2_, and RA area. The second model contained only the non-invasive variables of the first model (Table 7). C-statistic of the full model was 0.78 (0.64–0.92), and remained almost similar in the non-invasive version to 0.76 (0.62–0.90) (Figure 6).

**TABLE 7:** Variables used in pediatric risk stratification models.

Study	Model name	Definition of risk	Strata	Number of variables	WHO-FC	6MWD	NT-proBNP	RA area	Pericardial effusion	mRAP	CI	SvO_2_	PVR	WHO group 1 subgroup	Male age > 60	Systolic BP	Heart Rate	% predicted DL_*CO*_	ST2	TAPSE	Syncope	Height	BMI	mPAP/mSAP	PVRI	Acute vasoreactivity
Griffiths (44)	REVEAL 2.0 calculator	Risk score		11			or BNP																			
Griffiths (44)	REVEAL 2.0 calculator with REVEAL risk category	Risk category based on total score	5	11			or BNP																			
Griffiths (44)	REVEAL 2.0 calculator + ST2	Risk score		12			or BNP																			
Griffiths (44)	REVEAL 2.0 calculator with REVEAL risk category + ST2	Risk category based on total score	5	12			or BNP																			
Haarman (45)	WSPH 2013 model + SvO_2_ + RA area	#low risk criteria	2	13																						
Haarman (45)	WSPH 2013 non-invasive model + RA area	#low risk criteria	2	7																						

WHO-FC, WHO functional class; 6MWD, 6-minute walk distance; NT-proBNP, N-terminal-pro brain natriuretic peptide; RA area, right atrial area; mRAP mean right atrial pressure; CI, cardiac index; SvO_2_, mixed venous oxygen saturation; PVR, pulmonary vascular resistance; BP, blood pressure; DL_CO_, carbon monoxide lung diffusing capacity; ST2, soluble suppressor of tumorigenicity-2; TAPSE, tricuspid annular plane systolic excursion; BMI, body mass index; mPAP/mSAP, mean pulmonary arterial pressure/mean systemic arterial pressure ratio; PVRI, indexed PVR.

### Risk of bias

The PROBAST results of the ROB analysis are presented in Table 8. The ROB for the domains participants, predictors, and outcome was low for almost every study. However, many studies were judged as having a high ROB based on the described analysis, causing an overall high ROB for nearly all studies. To differentiate between studies scoring poorly on one or two signaling questions and those failing on nearly all aspects of the analysis, the judgment is marked with one, two or three asterixis. These asterixis correspond to respective one to three, four to six, and seven or more negatively answered questions (“no” or “no information”) out of nine for development studies and out of six for validation studies. There was low concern regarding applicability of models for participants, predictors, and outcome.

**TABLE 8:** PROBAST results.

		ROB								Applicability						Overall			
				
		Participants		Predictors		Outcome		Analysis		Participants		Predictors		Outcome		ROB		Applicability	
										
Study	Model name	dev	val	dev	val	dev	val	dev	val	dev	val	dev	val	dev	val	dev	val	dev	val
Anderson (53)	REVEAL 2.0 calculator		–		+		+		–*		+		+		+		–		+
Austin (37)	REVEAL risk score calculator		+		+		+		–*		+		+		+		–		+
	Echocardiographic approach	+	+	+	+	+	+	–**	–*	+	+	+	+	+	+	–	–	+	+
Benza (10)	REVEAL risk score calculator	+	+	+	+	+	+	–**	–*	+	+	+	+	+	+	–	–	+	+
Benza (18)	REVEAL 2.0 calculator	+		+		+		–**		+		+		+		–		+	
	COMPERA model		+		+		+		–*		+		+		+		–		+
	FPHR invasive model		+		+		+		–*		+		+		+		–		+
Benza (19)	REVEAL Lite 2	+		+		+		–**		+		+		+		–		+	
Boucly (23)	FPHR invasive model	–		+		+		–***		+		+		+		–		+	
	FPHR non-invasive model	–		+		+		–***		+		+		+		–		+	
Boucly (42)	COMPERA model (abbreviated)		–		+		+		–*		+		+		+		–		+
	COMPERA 2.0 model		–		+		+		–*		+		+		+		–		+
Chang (54)	REVEAL Lite 2		+		+		+		–*		+		+		+		–		+
	COMPERA model		+		+		+		–*		+		+		+		–		+
	FPHR invasive model		+		+		+		–*		+		+		+		–		+
Chiu (24)	ESC/ERS 2015 model (abbreviated)	+		+		+		–***		+		+		+		–		+	
Dardi (25)	COMPERA model		+		+		+		–*		+		+		+		–		+
	FPHR invasive model		+		+		+		–*		+		+		+		–		+
	ESC/ERS 2015 model (abbreviated)	+		+		+		–**		+		+		+		–		+	
Ghio (38)	Echocardiographic approach	+		+		+		–**		+		+		+		–		+	
Griffiths (44)	REVEAL 2.0 calculator	–			+		+		–**		+		+		+		–		+
	REVEAL 2.0 calculator + ST2	–		+		+		–***		+		+		+		–		+	
Gong (55)	COMPERA model		+		+		+		–*		+		+		+		–		+
Haarman (45)	WSPH 2013 model + SvO_2_ + RA area	+		+		+		–**		+		+		+		–		+	
	WSPH 2013 non-invasive model + RA area	+		+		+		–**		+		+		+		–		+	
Haddad (41)	Eigenvector centrality model	+		+		+		–**		+		+		+		–		+	
	Eigenvector centrality model–imaging-focused	+		+		+		–**		+		+		+		–		+	
	Eigenvector centrality model–biomarker-focused	+		+		+		–**		+		+		+		–		+	
Harbaum (43)	REVEAL 2.0 calculator		+		+		+		–*		+		+		+		–		+
	COMPERA model		+		+		+		–*		+		+				–		+
	FPHR non-invasive model		+		+		+		–*		+		+				–		+
	REVEAL 2.0 calculator + PaCO_2_	+		+		+		–**		+		+		+		–			
	COMPERA model + PaCO_2_	+		+				–**		+		+		+		–		+	
	FPHR non-invasive model + PaCO_2_	+		+		+		–**		+		+		+		–		+	
Hjalmarsson (56)	SPAHR model		+		+		+		–*		+		+		+		–		+
Hoeper (20)	COMPERA model	+		+		+		–**		+		+		+		–		+	
Hoeper (22)	COMPERA model (abbreviated)	–		+		+		–***		+		+		+		–		+	
	COMPERA 2.0	–		+		+		–***		+		+		+		–		+	
Imai (26)	ESC/ERS 2015 model (abbreviated)	+		+		+		–***		+		+		+		–		+	
Kanwar (19)	Tree-augmented naïve Bayes model of REVEAL 2.0	+	+	+	+	+	+	–**	–*	+	+	+	+	+	+	–	–	+	+
Kylhammar (64)	SPAHR model	+		+		+		–**		+		+		+		–		+	
Kylhammar (56)	SPAHR model		–		+		+		–*		+		+		+		–		+
Lee (52)	Scottish composite score	+	+	+	+	+	+	–**	–**	+	+	+	+	+	+	–	–	+	+
Lewis (34)	REVEAL 2.0 calculator		+		+		+	–*			+		+		+	–			+
	FPHR invasive model ISWD	+		+		+			–**	+		+		+			–	+	
	REVEAL 2.0 calculator + RVESVi	+		+		+			–**	+		+		+			–	+	
	FPHR invasive model ISWD + RVESVi	+		+		+			–**	+		+		+			–	+	
Lewis (62)	REVEAL 2.0 calculator ISWD	+		+		+		–*		+		+		+		–		+	
	FPHR invasive model ISWD	+		+		+		–**		+		+		+		–		+	
Li (27)	ESC/ERS 2015 model (abbreviated)	+		+		+		–***		+		+		+		–		+	
Mercurio (28)	FPHR invasive model		+		+		+		–**		+		+		+		–		+
	ESC/ERS 2015 model (abbreviated)	+		+		+		–***		+		+		+		–		+	
Mullin (58)	REVEAL risk score calculator		+		+		+		–*		+		+		+		–		+
Qu (59)	REVEAL risk score calculator		+		+		+		–*		+		+		+		–		+
Quan (46)	REVEAL 2.0 calculator		+		+		+		+		+		+		+		+		+
	REVEAL Lite 2		+		+		+		–*		+		+		+		–		+
	COMPERA model		+		+		+		+		+		+		+		+		+
	FPHR invasive model		+		+		+		–*		+		+		+		–		+
	FPHR non-invasive model		+		+		+		–*		+		+		+		–		+
Rhodes (39)	Plasma proteome 6	+	+	+	+	+	+	–*	–*	+	+	+	+	+	+	–	–	+	+
	Plasma proteome 6 + NT-proBNP	+	+	+	+	+	+	–*	–*	+	+	+	+	+	+	–	–	+	+
Simpson (35)	REVEAL 2.0 calculator		–		+		+		–*		+		+		+		–		+
	FPHR invasive model		–		+		+		–*		+		+		+		–		+
	FPHR non-invasive model		–		+		+		–*		+		+		+		–		+
	REVEAL 2.0 calculator + endostatin	–		+		+		–**		+		+		+		–		+	
	FPHR invasive model + NTproBNP	–		+		+		–**		+		+		+		–		+	
	FPHR invasive model + endostatin	–		+		+		–**		+		+		+		–		+	
	FPHR invasive model + NTproBNP + endostatin	–		+		+		–**		+		+		+		–		+	
	FPHR non-invasive model + endostatin	–		+		+		–**		+		+		+		–		+	
Sitbon (60)	REVEAL risk score calculator		+		+		+		–*		+		+		+		–		+
Vicenzi (63)	COMPERA model		–		+		+		–**		+		+		+		–		+
	FPHR invasive model		–		+		+		–**		+		+		+		–		+
	FPHR non-invasive model		–		+		+		–**		+		+		+		–		+
	COMPERA model + TAPSE/TRV or TAPSE/sPAP	–		+		+		–***		+		+		+		–		+	
	FPHR invasive model + TAPSE/TRV or TAPSE/sPAP	–		+		+		–***		+		+		+		–		+	
	FPHR non-invasive model + TAPSE/TRV or TAPSE/sPAP	–		+		+		–***		+		+		+		–		+	
Vraka (61)	REVEAL 2.0 calculator		+		+		+		–*		+		+		+		–		+
	COMPERA model		+				+		–*		+		+		+		–		+
	FPHR invasive model		+		+		+		–*		+		+		+		–		+
Wang (29)	ESC/ERS 2015 model (abbreviated)	+		+		+		–***		+		+		+		–		+	
Weatherald (48)	FPHR invasive model		+		+		+		–*		+		+		+		–		+
Xanthouli (30)	COMPERA model		+		+		+		–**		+		+		+		–		+
	FPHR non-invasive model		+		+		+		–**		+		+		+		–		+
	ESC/ERS 2015 model (abbreviated, non-invasive)	+		+		+		–***		+		+		+		–		+	
Xiong (31)	REVEAL risk score calculator		–		+		+		–*		+		+		+		–		+
	ESC/ERS 2015 model (abbreviated, non-invasive)	–		+		+		–**		+		+		+		–		+	
Yogeswaran (32)	ESC/ERS 2015 model (abbreviated)	+		+		+		–**		+		+		+		–		+	
	ESC/ERS 2015 model (abbreviated) + TAPSE/sPAP	+		+		+		–**		+		+		+		–		+	
Yogeswaran (40)	COMPERA model		+		+		+		–*		+		+		+		–		+
	Biomarker approach	+	+	+	+	+	+	–**	–*	+	+	+	+	+	+	–	–	+	+
Zelt (33)	REVEAL risk score calculator		+		+		+		–*		+		+		+		–		+
	REVEAL 2.0 calculator		+		+		+		–*		+		+		+		–		+
	FPHR invasive model		+		+		+		–*		+		+		+		–		+
	ESC/ERS 2015 model (abbreviated)	+		+		+		–**		+		+		+		–		+	
	REVEAL risk score calculator + eGFR		+		+		+	–**			+		+		+	–			+
	FPHR invasive model + eGFR		+		+		+	–**			+		+		+	–			+
	ESC/ERS 2015 model (abbreviated) + eGFR	+		+		+		–**		+		+		+		–		+	

PROBAST, Prediction model Risk Of Bias Assessment Tool; ROB, risk of bias; dev, development; val, validation; ST2, soluble suppressor of tumorigenicity-2; SvO_2_, mixed venous oxygen saturation; RA area, right atrial area; PaCO_2_, arterial carbon dioxide partial pressure; ISWD, incremental shuttle walk distance; RVESVi, right ventricular end-systolic volume index; NT-proBNP, N-terminal-pro brain natriuretic peptide; TAPSE/TRV, tricuspid annular plane systolic excursion/tricuspid regurgitation velocity ratio; TAPSE/sPAP, tricuspid annular plane systolic excursion/systolic pulmonary artery pressure ratio; eGFR, estimated glomerular filtration rate. +Indicates low ROB/low concern regarding applicability; –indicates high ROB/high concern regarding applicability; *1–3, **4–6, ***≥ 7 negatively answered questions.

## Discussion

In this systematic review we identified twenty different risk stratification models that have been proposed for adult PAH and only two for pediatric PAH. The REVEAL risk calculators are the most frequently validated models in literature, followed by the COMPERA model and FPHR invasive and non-invasive models. For the enhancement of existing risk stratification models, the FPHR invasive method and REVEAL 2.0 calculator have been studied most frequently. The non-invasive WHO-FC, 6MWD, and BNP/NT-proBNP, and the invasive mRAP, CI, and SvO_2_ were found to be the variables that are most often used for the risk stratification of PAH. Reported c-statistics representing model predictive strength range from 0.39 to 0.77. Studies enhancing models by adding new variables report improvement of model strength.

Most risk stratification models include the non-invasive variables of WHO-FC, 6MWD, and BNP/NT-proBNP. The inclusion of these parameters in risk stratification may be due to the extensive studies on the prognostic value of these parameters, and stresses their important prognostic abilities in adult PAH patients. Based on the comparable predictive strength of non-invasive models and models including invasive parameters reported in three studies (19, 45, 46), a fully non-invasive risk stratification may be feasible. However, data supporting fully non-invasive risk stratification models are still scarce. Therefore, it may still be too early to set aside the invasive parameters included in most risk stratification models.

In the identified risk stratification models, different methods are used to combine cut-off scores of individual variables to determine the overall risk status. The three main definitions of risk are (1) the number of low risk criteria, (2) risk category based on an average score, and (3) risk category based on the total sum of the score. Furthermore, the risk stratification models can use weighted or unweighted variables. Risk stratification models using the number of low risk criteria (e.g., FPHR invasive and non-invasive method) or an average score (e.g., COMPERA and SPAHR models) do not take the weight of the variables into account for their determination of risk. This may lead to an underestimation or overestimation of patient risk. The REVEAL risk calculators were the only models found to consider the weighted values for individual variables in the calculation of risk. Variables that showed at least a twofold increase in hazard for mortality according to the multivariable Cox proportional hazard model were assigned two points, whereas variables with lower hazard received one point (10). This inclusion of variable weight in the risk estimation does appear to have an effect on the discriminatory ability of the model. C-statistics found in studies using the REVEAL risk calculators were, in general for most studies, higher (0.70–0.75) than those reported for COMPERA and FPHR models (0.62–0.69). These findings may favor the use of weighted risk scores instead of averages or number of low or high risk criteria in further development of risk stratification models.

Overall, the c-statistic of most studies was found to range between 0.6 and 0.8. Considering that a c-statistic of 0.5 indicates a poor prediction and 1.0 a perfect prediction, we may consider the current risk stratification models to have a moderate predictive ability. Whether or not this is sufficient enough to rely on for optimal treatment strategies can be debated. In the recently released 2022 ESC/ERS guidelines for the diagnosis and treatment of PH (4), the four-strata COMPERA 2.0 model of Hoeper et al. (22) using WHO-FC, 6MWD, and BNP/NT-proBNP is recommended for risk stratification at follow-up to guide treatment strategies in adult patients with PAH. The c-statistic for 1 year mortality of this four-strata model was reported to be 0.67 at baseline and 0.73 at follow-up, in an external validation study by Boucly et al. (42). According to this, the authors would advocate that we should strive for improving current risk stratification models.

A possible approach for improving risk stratification models may be the addition of new parameters. The increase of the c-statistic in all enhancement studies, except for the addition of arterial carbon dioxide partial pressure to the FPHR non-invasive model (43), shows that the predictive strength of risk stratification models can be improved by adding imaging or serum biomarkers. Of all the enhancement studies, the addition of the right ventricular end-systolic volume index seems most promising (34). Prospective and external validation studies are needed to further establish the predictive value of enhanced models.

Furthermore, the use of risk stratification is not restricted to estimate risk at diagnosis or initiation of therapy. Also serial risk stratification every 3–6 months is proposed in order to use follow-up risk estimates to evaluate treatment response and to identify the need to escalate therapy (47). Recent reports show that risk stratifications may have a better discrimination of outcome at first follow-up RHC compared to baseline (48), and that changes in risk status are predictive of survival (49). Moreover, the addition of serial changes in NT-proBNP or right heart reverse remodeling (a combination of three echocardiographic parameters) increased the c-statistic of respective the eigenvector centrality model of Haddad et al. (41) (0.81–0.85) and the REVEAL 2.0 calculator (0.69–0.87) (50). As such the strength of risk stratification models may lie in serial assessments.

Data regarding the use of risk stratification models in pediatric PAH is extremely scarce. In this review, only two pediatric PAH studies were found, one based on the variables recommended by the WSPH 2013 pediatric task force and one based on the REVEAL 2.0 calculator. Nonetheless, risk stratification to guide treatment strategies is currently recommended also in the pediatric population. The updated guideline of the European Pediatric Pulmonary Vascular Disease for the diagnosis and treatment of pediatric pulmonary hypertension presents a risk score sheet for pediatric PH based solely on expert opinion (51). However, no validation yet exists and in the guideline it is stated that it is not clear which cut-offs should be used for the risk stratification variables. For this reason, Haarman et al. (45) in their study used cut-off values derived from separate prognosticator studies in children with PAH. Considering the reference class problem, which dictates that the prediction for the individual patient depends on the reference class the patient is assigned to, it is recommended to develop a risk stratification model with variable cut-offs and weights designed specifically for the pediatric population.

Nearly all studies included in this systematic review were judged to have a high ROB based on their analysis. This can be explained with closer observation of the analysis domain of PROBAST (16), the tool that was used to rate ROB. First, according to PROBAST, the number of events (death or death + transplant) per variable should be higher than 10 for development studies, and for validation studies at least 100 participants with the outcome are required. These criteria were met by approximately only half of the included adult studies. Since pediatric PAH is a rare disease, none of the included pediatric studies met the criteria for the number of participants, which shows the limitation of the applicability of PROBAST in a rare disease. Secondly, if continuous variables were dichotomized or categorized for the development of a model, according to PROBAST the model could have a high ROB. However, categorization forms the basis of risk stratification and thus many model development were rated to have a high ROB. For validation studies categorization of continuous variables was allowed if the cut-offs were similar to the original model. Third aspect in PROBAST is the inclusion of all enrolled participants in the analysis and the appropriate handling of missing data, since excluding patients with missing data may cause selection bias. Besides, selection bias is a reasonable risk of registry studies since there are nearly always missing data due to the data not being collected according to a protocol or for the research question at hand. Fourth, multiple studies did not report c-statistic or AUROC, where PROBAST demands reporting of both calibration and discrimination measures. Information on model overfitting and optimism in model performance was also often not described. Finally, the weights of the variables in the final model had to correspond to the results from the reported multivariable analysis. As discussed earlier, models defining risk by the number of low risk criteria or based on an average score do not take the weight of a variable into account, and thus these studies were also at high ROB.

This study has several limitations. Not all included studies reported a c-statistic, which may have caused a bias in the judgment of the prognostic value of the models. The patients included in studies performed more recently were receiving treatment according to the risk stratification-based treatment algorithms. This may have influenced the outcome of those patients, which could have affected the prognostic value of risk stratification models of these studies. No meta-analysis was performed limiting direct conclusions on which model performs best. In order to keep focus, the studies concerning serial risk stratification were disregarded, limiting the ability to discuss the value of serial follow-up risk stratification.

For future purposes, it is recommended to perform prospective validation studies of the risk stratification models since now only retrospective studies of risk stratification exist. Studies developing new models or validating existing models should consider including both calibration and discrimination measures as both are needed to thoroughly describe the performance of the model. Furthermore, an individual patient data systematic review is recommended to define which risk stratification model has the best performance.

## Conclusion

This systematic review contributes to our current knowledge on risk stratification in PAH and emphasizes the very limited presence of studies reporting risk stratification in pediatric PAH. The variables found to be used the most frequently in risk stratification models are WHO-FC, 6MWD, NT-proBNP (or BPN), mRAP, CI, and SvO_2_. The prognostic value of current risk stratification models is moderate to good, at best, and may be improved by adding new imaging and serum biomarkers, using weighted risk stratification variables, and adding changes in clinical parameters at serial risk stratification during follow-up. Moreover, there is a need for prospective validation of risk stratification models and more research into risk stratification for pediatric PAH has to be pursued.

## Data availability statement

The raw data supporting the conclusions of this article will be made available by the authors, without undue reservation.

## Author contributions

All authors contributed to the development of the research question and selection criteria. CL and SW developed the search strategy and drafted the initial manuscript. Title-abstract and full-text screening was performed by CL and JD, where RB was consulted in case of disagreement. Data extraction, risk of bias, and data analysis was performed by CL in consultation with RB and JD. RB and JD reviewed and revised the manuscript.
