# Supplementary material for: Risk stratification in adult and pediatric pulmonary arterial hypertension: A systematic review
Source: Front Cardiovasc Med. 2022 Nov 10;9:1035453. doi: 10.3389/fcvm.2022.1035453 (PMC9684185; doi:10.3389/fcvm.2022.1035453)
Supplement: Supplementary file 1 [file Table_1.DOCX]

Supplementary Material

Supplementary Table 1. Search strategy

|  | **PubMed** | **Embase** | **Web of Science** |
| --- | --- | --- | --- |
| **Search string** | ("Familial Primary Pulmonary Hypertension"[MeSH] OR "Pulmonary Arterial Hypertension"[MeSH] OR "pulmonary arterial hypertension"[tiab] OR "pulmonary artery hypertension"[tiab] OR "primary pulmonary hypertension"[tiab] OR "pediatric pulmonary hypertension"[tiab] OR "paediatric pulmonary hypertension"[tiab] OR  (("Hypertension, Pulmonary"[Mesh] OR "pulmonary hypertension"[tiab]) *AND* ("Child"[Mesh] OR "Adolescent"[Mesh] OR "Infant"[Mesh] OR "Pediatrics"[Mesh] OR child*[tiab] OR pediatric*[tiab] OR paediatric*[tiab] OR infan*[tiab] OR baby[tiab] OR babies[tiab] OR neonat*[tiab] OR kids[tiab] OR adolescen*[tiab] OR newborn*[tiab])))  AND  ("Risk Assessment"[Mesh] OR (risk[tiab] *AND* stratif*[tiab]) OR "risk assess*"[tiab] OR "risk model*"[tiab] OR "risk tool*"[tiab] OR "risk profil*"[tiab] OR “risk factor profil*”[tiab] OR "prediction model*"[tiab] OR "predictive model*"[tiab] OR “prognostic model*” OR "prediction tool*"[tiab] OR "predictive tool*"[tiab] OR "risk algorithm*"[tiab] OR "risk equation*"[tiab] OR "risk calculat*"[tiab] OR "risk formula"[tiab] OR “risk predict*”[tiab])  NOT  (("Animals"[Mesh] NOT "Humans"[Mesh]) OR "Review" [pt] OR "Case Reports" [pt]) | (("pulmonary arterial hypertension" OR "pulmonary artery hypertension" OR "primary pulmonary hypertension" OR "pediatric pulmonary hypertension" OR "paediatric pulmonary hypertension"):ab,ti,kw OR (('pulmonary hypertension'/exp OR "pulmonary hypertension":ab,ti,kw) *AND* ('child'/exp OR 'adolescent'/exp OR (child* OR pediatric OR paediatric* OR infan* OR baby OR babies OR neonat* OR kids OR adolescen* OR newborn*):ab,ti,kw)))  AND  ('risk stratification'/exp OR 'risk assessment'/exp OR 'risk algorithm'/exp OR ((risk AND stratif*) OR ‘risk assess*’ OR ‘risk tool*’ OR ‘prognostic model*’ OR (predict* NEXT/3 model*) OR (risk NEXT/3 predict*) OR (risk NEXT/5 (model* OR profil* OR algorithm* OR equation* OR calculat* OR formula))):ab,ti,kw)  NOT  (('animal'/exp NOT 'human'/exp) OR 'review'/de OR 'case report'/exp OR 'conference abstract'/it) | TS=("pulmonary arterial hypertension" OR "pulmonary artery hypertension" OR "primary pulmonary hypertension" OR "pediatric pulmonary hypertension" OR "paediatric pulmonary hypertension" OR (("pulmonary hypertension") *AND* (child* OR pediatric OR paediatric* OR infan* OR baby OR babies OR neonat* OR kids OR adolescen* OR newborn*)))  AND  TS=(("risk" *AND* "stratif*") OR "risk assess*" OR "risk model*" OR "risk tool*" OR "risk profil*" OR “risk factor profil*” OR “prediction model*” OR “predictive model*” OR “prognostic model*” OR "prediction tool*" OR "predictive tool*" OR “risk algorithm*” OR “risk equation*” OR “risk calculat*” OR “risk formula” OR “risk predict*”) NOT  DT=("review" OR "meeting abstract") |
| **Number of hits on June 8^th^ 2022** | 766 | 1083 | 546 |
