# Supplementary material for: Risk stratification in adult and pediatric pulmonary arterial hypertension: A systematic review
Source: Front Cardiovasc Med. 2022 Nov 10;9:1035453. doi: 10.3389/fcvm.2022.1035453 (PMC9684185; doi:10.3389/fcvm.2022.1035453)
Supplement: Supplementary file 2 [file Table_2.DOCX]

Supplementary Material

Supplementary Table 2. Studies excluded during full text review.

| **First author** | **Journal (abbreviated)** | **Year** | **Exclusion reason** |
| --- | --- | --- | --- |
| Gruss (1) | Revista Medica Del Uruguay | 2019 | Not in English |
| Stubbe (2) | Pneumologie | 2022 | Not in English |
| Fernandes (3) | Pulmonary Circulation | 2021 | Case series |
| Abman (4) | Archives of Disease in Childhood: Fetal and Neonatal Edition | 2002 | Review |
| Feinstein (5) | Seminars in Thoracic and Cardiovascular Surgery: Pediatric Cardiac Surgery Annual | 2009 | Review |
| Keogh (6) | Journal of the American College of Cardiology | 2009 | Review |
| Hooper (7) | Vascular Pharmacology | 2007 | Review |
| Balkin (8) | Pediatr Crit Care Med | 2018 | Not PAH or PH respiratory |
| Garcia (9) | Respir Med | 2021 | Not PAH or PH respiratory |
| Gary (10) | Thromb Res | 2012 | Not PAH or PH respiratory |
| Geenen (11) | Am Heart J | 2019 | Not PAH or PH respiratory |
| Spilimbergo (12) | Arq Bras Cardiol | 2022 | Not PAH or PH respiratory |
| Woo (13) | Pediatric Pulmonology | 2004 | Not PAH or PH respiratory |
| Yaylali (14) | Anatol J Cardiol | 2019 | Not PAH or PH respiratory |
| Abenhaim (15) | Chest | 1994 | No risk stratification model |
| Alandejani (16) | Front Cardiovasc Med | 2022 | No risk stratification model |
| Amsallem (17) | Circ Cardiovasc Imaging | 2017 | No risk stratification model |
| Benza (18) | Circulation | 2010 | No risk stratification model |
| Benza (19) | Transplantation | 2010 | No risk stratification model |
| Benza (20) | J Heart Lung Transplant | 2021 | No risk stratification model |
| Chin (21) | Circulation | 2019 | No risk stratification model |
| Cogswell (22) | J Heart Lung Transplant | 2012 | No risk stratification model |
| Cogswell (23) | J Heart Lung Transplant | 2014 | No risk stratification model |
| Deng (24) | Clin Respir J | 2019 | No risk stratification model |
| Gauthier-Loiselle (25) | Curr Med Res Opin | 2022 | No risk stratification model |
| Gomberg-Maitland (26) | J Heart Lung Transplant | 2013 | No risk stratification model |
| Guo (27) | Hypertension | 2020 | No risk stratification model |
| Guo (28) | European Heart Journal Cardiovascular Imaging | 2021 | No risk stratification model |
| Haddad (29) | JACC Cardiovasc Imaging | 2015 | No risk stratification model |
| Hsu (30) | Am Heart J | 2011 | No risk stratification model |
| Humbert (31) | Eur Respir J | 2010 | No risk stratification model |
| Kawamukai (32) | Heart Vessels | 2019 | No risk stratification model |
| Kempny (33) | Circulation | 2017 | No risk stratification model |
| Lai (34) | Front Cardiovasc Med | 2022 | No risk stratification model |
| Law (35) | Am Heart J | 2007 | No risk stratification model |
| Lewis (36) | Eur Respir J | 2021 | No risk stratification model |
| Ling (37) | American Journal of Respiratory and Critical Care Medicine | 2012 | No risk stratification model |
| Liu (38) | Int J Cardiovasc Imaging | 2020 | No risk stratification model |
| Mazimba (39) | Respir Med | 2016 | No risk stratification model |
| Mazimba (40) | Heart Lung Circ | 2019 | No risk stratification model |
| Mwansa (41) | Clin Cardiol | 2017 | No risk stratification model |
| Nickel (42) | Eur Respir J | 2012 | No risk stratification model |
| Prins (43) | J Am Heart Assoc | 2020 | No risk stratification model |
| Qaderi (44) | J Clin Med | 2021 | No risk stratification model |
| Rehman (45) | Int J Cardiol | 2018 | No risk stratification model |
| Rhodes (46) | Lancet Respir Med | 2017 | No risk stratification model |
| Ruth (47) | Heart Lung Circ | 2019 | No risk stratification model |
| Siddiqui (48) | Am J Cardiol | 2018 | No risk stratification model |
| Sandoval (49) | Circulation | 1994 | No risk stratification model |
| Thenappan (50) | Eur Respir J | 2010 | No risk stratification model |
| Thenappan (51) | Chest | 2012 | No risk stratification model |
| van der Bruggen (52) | Chest | 2021 | No risk stratification model |
| Arvanitaki (53) | Pulm Circ | 2019 | Not eligible endpoint |
| Badagliacca (54) | J Heart Lung Transplant | 2016 | Not eligible endpoint |
| Badagliacca (55) | J Heart Lung Transplant | 2022 | Not eligible endpoint |
| Bouzina (56) | Scand Cardiovasc J | 2021 | Not eligible endpoint |
| Grignola (57) | Front Physiol | 2021 | Not eligible endpoint |
| Hoffmann-Vold (58) | Arthritis Rheumatol | 2018 | Not eligible endpoint |
| Hrustanovic-Kadic (59) | Annals of the American Thoracic Society | 2021 | Not eligible endpoint |
| Min (60) | Am J Respir Crit Care Med | 2021 | Not eligible endpoint |
| Sahay (61) | PLoS One | 2020 | Not eligible endpoint |
| Sahay (62) | Pulm Circ | 2022 | Not eligible endpoint |
| Scelsi (63) | Diagnostics (Basel) | 2020 | Not eligible endpoint |
| Simons (64) | Adv Ther | 2019 | Not eligible endpoint |
| Stolfo (65) | J Am Soc Echocardiogr | 2020 | Not eligible endpoint |
| Cornwell (66) | Chest | 2011 | No appropriate statistics |
| Ablonczy (67) | Transplant Proc | 2021 | PAH not confirmed with RHC |
| Vlachou (68) | Rheumatology (United Kingdom) | 2022 | PAH not confirmed with RHC |
| Boucly (69) | Eur Respir J | 2018 | Other |
| Clerico (70) | Clin Sci (Lond) | 2010 | Other |
| Hjalmarsson (71) | Eur Respir J | 2018 | Other |
| Kissoon (72) | Journal of Critical Care | 2006 | Other |
| Olsson (73) | Eur Respir J | 2022 | Other |
| Packer (74) | Chest | 1989 | Other |
| Rubin (75) | ACC Cardiosource Review Journal | 2006 | Other |
| Smith (76) | Crit Care Med | 1999 | Other |
| Valentin (77) | Eur Respir J | 2021 | Other |
| Warnes (78) | ACC Cardiosource Review Journal | 2006 | Other |
| Wilson (79) | Pulm Circ | 2020 | Other |
| Xiong (80) | Pulm Circ | 2018 | Other |

# Supplementary references

1. Gruss AI, Pascal G, Chao C, Janssen B, Bedo C, Salisbury JP, et al. Ten Years of Experience of a Reference Center in Pulmonary Arterial Hypertension in Uruguay. *Revista Medica Del Uruguay* (2019) 35(3):193-202. doi: 10.29193/Rmu.35.3.3.

2. Stubbe B, Halank M, Seyfarth HJ, Obst A, Desole S, Opitz CF, et al. Risk Stratification in Patients with Pulmonary Arterial Hypertension under Treatment - Results of Four German Centers. *Pneumologie* (2022) 76(5):330-9.

3. Fernandes C, da Silva TAF, Alves JL, Jr., Jardim CVP, de Souza R. Inhaled Iloprost as Third Add-on Therapy in Idiopathic Pulmonary Arterial Hypertension. *Pulm Circ* (2021) 11(1):2045894020981350. Epub 2021/02/04. doi: 10.1177/2045894020981350.

4. Abman SH. Monitoring Cardiovascular Function in Infants with Chronic Lung Disease of Prematurity. *Arch Dis Child Fetal Neonatal Ed* (2002) 87(1):F15-8. Epub 2002/07/02. doi: 10.1136/fn.87.1.f15.

5. Feinstein JA. Evaluation, Risk Stratification, and Management of Pulmonary Hypertension in Patients with Congenital Heart Disease. *Semin Thorac Cardiovasc Surg Pediatr Card Surg Annu* (2009) 12(1):106-11. Epub 2009/04/08. doi: 10.1053/j.pcsu.2009.01.010.

6. Keogh AM, Mayer E, Benza RL, Corris P, Dartevelle PG, Frost AE, et al. Interventional and Surgical Modalities of Treatment in Pulmonary Hypertension. *J Am Coll Cardiol* (2009) 54(1 Suppl):S67-S77. Epub 2009/07/09. doi: 10.1016/j.jacc.2009.04.016.

7. Hooper WC, Mensah GA, Haworth SG, Black SM, Garcia JG, Langleben D. Vascular Endothelium Summary Statement V: Pulmonary Hypertension and Acute Lung Injury: Public Health Implications. *Vascul Pharmacol* (2007) 46(5):327-9. Epub 2007/01/02. doi: 10.1016/j.vph.2006.10.017.

8. Balkin EM, Zinter MS, Rajagopal SK, Keller RL, Fineman JR, Steurer MA. Intensive Care Mortality Prognostic Model for Pediatric Pulmonary Hypertension. *Pediatr Crit Care Med* (2018) 19(8):733-40. Epub 2018/06/21. doi: 10.1097/PCC.0000000000001636.

9. Garcia MVF, Souza R, Costa ELV, Fernandes C, Jardim CVP, Caruso P. Outcomes and Prognostic Factors of Decompensated Pulmonary Hypertension in the Intensive Care Unit. *Respir Med* (2021) 190:106685. Epub 2021/11/26. doi: 10.1016/j.rmed.2021.106685.

10. Gary T, Starz I, Belaj K, Steidl K, Hafner F, Froehlich H, et al. Hyperlipidemia Is Associated with a Higher Pulmonary Artery Systolic Pressure in Patients after Pulmonary Embolism. *Thromb Res* (2012) 129(1):86-8. Epub 2011/10/14. doi: 10.1016/j.thromres.2011.09.018.

11. Geenen LW, Baggen VJM, Koudstaal T, Boomars KA, Eindhoven JA, Boersma E, et al. The Prognostic Value of Various Biomarkers in Adults with Pulmonary Hypertension; a Multi-Biomarker Approach. *Am Heart J* (2019) 208:91-9. Epub 2018/12/24. doi: 10.1016/j.ahj.2018.11.001.

12. Spilimbergo FB, Assmann TS, Bellon M, Hoscheidt LM, Caurio CFB, Puchalski M, et al. Soluble Guanylate Cyclase Stimulators (Riociguat) in Pulmonary Hypertension: Data from Real-Life Clinical Practice in a 3-Year Follow-Up. *Arq Bras Cardiol* (2022).

13. Woo MS. Living Related Donors. *Pediatr Pulmonol Suppl* (2004) 26:114-5. Epub 2004/03/20. doi: 10.1002/ppul.70074.

14. Yaylali YT, Basarici I, Kilickiran Avci B, Meric M, Sinan UY, Senol H, et al. Risk Assessment and Survival of Patients with Pulmonary Hypertension: Multicenter Experience in Turkey. *Anatol J Cardiol* (2019) 21(6):322-30. Epub 2019/05/31. doi: 10.14744/AnatolJCardiol.2019.53498.

15. Abenhaim L, Moride Y, Rich S, Chaslerie A, Brenot F, Higenbottam T, et al. The International Primary Pulmonary-Hypertension Study (Ipphs). *Chest* (1994) 105(2):S37-S41.

16. Alandejani F, Hameed A, Tubman E, Alabed S, Shahin Y, Lewis RA, et al. Imaging and Risk Stratification in Pulmonary Arterial Hypertension: Time to Include Right Ventricular Assessment. *Front Cardiovasc Med* (2022) 9:797561.

17. Amsallem M, Sweatt AJ, Aymami MC, Kuznetsova T, Selej M, Lu H, et al. Right Heart End-Systolic Remodeling Index Strongly Predicts Outcomes in Pulmonary Arterial Hypertension: Comparison with Validated Models. *Circ Cardiovasc Imaging* (2017) 10(6). Epub 2017/06/09. doi: 10.1161/CIRCIMAGING.116.005771.

18. Benza RL, Miller DP, Gomberg-Maitland M, Frantz RP, Foreman AJ, Coffey CS, et al. Predicting Survival in Pulmonary Arterial Hypertension: Insights from the Registry to Evaluate Early and Long-Term Pulmonary Arterial Hypertension Disease Management (Reveal). *Circulation* (2010) 122(2):164-72. Epub 2010/06/30. doi: 10.1161/CIRCULATIONAHA.109.898122.

19. Benza RL, Miller DP, Frost A, Barst RJ, Krichman AM, McGoon MD. Analysis of the Lung Allocation Score Estimation of Risk of Death in Patients with Pulmonary Arterial Hypertension Using Data from the Reveal Registry. *Transplantation* (2010) 90(3):298-305.

20. Benza RL, Ghofrani HA, Grunig E, Hoeper MM, Jansa P, Jing ZC, et al. Effect of Riociguat on Right Ventricular Function in Patients with Pulmonary Arterial Hypertension and Chronic Thromboembolic Pulmonary Hypertension. *J Heart Lung Transplant* (2021) 40(10):1172-80. Epub 2021/08/07. doi: 10.1016/j.healun.2021.06.020.

21. Chin KM, Rubin LJ, Channick R, Di Scala L, Gaine S, Galie N, et al. Association of N-Terminal Pro Brain Natriuretic Peptide and Long-Term Outcome in Patients with Pulmonary Arterial Hypertension. *Circulation* (2019) 139(21):2440-50. Epub 2019/04/16. doi: 10.1161/CIRCULATIONAHA.118.039360.

22. Cogswell R, Kobashigawa E, McGlothlin D, Shaw R, De Marco T. Validation of the Registry to Evaluate Early and Long-Term Pulmonary Arterial Hypertension Disease Management (Reveal) Pulmonary Hypertension Prediction Model in a Unique Population and Utility in the Prediction of Long-Term Survival. *J Heart Lung Transplant* (2012) 31(11):1165-70. Epub 2012/10/16. doi: 10.1016/j.healun.2012.08.009.

23. Cogswell R, Pritzker M, De Marco T. Performance of the Reveal Pulmonary Arterial Hypertension Prediction Model Using Non-Invasive and Routinely Measured Parameters. *J Heart Lung Transplant* (2014) 33(4):382-7. Epub 2014/02/19. doi: 10.1016/j.healun.2013.12.015.

24. Deng X, Jin B, Li S, Li Y, Zhou H, Wu Y, et al. Guideline Implementation and Early Risk Assessment in Pulmonary Arterial Hypertension Associated with Congenital Heart Disease: A Retrospective Cohort Study. *Clin Respir J* (2019) 13(11):693-9. Epub 2019/08/17. doi: 10.1111/crj.13076.

25. Gauthier-Loiselle M, Tsang Y, Lefebvre P, Agron P, Royer J, Bell Lynum KS, et al. Development and Evaluation of a Predictive Algorithm for Unsatisfactory Response among Patients with Pulmonary Arterial Hypertension Using Health Insurance Claims Data. *Curr Med Res Opin* (2022):1-12. Epub 2022/03/05. doi: 10.1080/03007995.2022.2049162.

26. Gomberg-Maitland M, Glassner-Kolmin C, Watson S, Frantz R, Park M, Frost A, et al. Survival in Pulmonary Arterial Hypertension Patients Awaiting Lung Transplantation. *J Heart Lung Transplant* (2013) 32(12):1179-86. Epub 2013/10/01. doi: 10.1016/j.healun.2013.08.016.

27. Guo X, Lai J, Wang H, Tian Z, Zhao J, Li M, et al. Predictive Value of Pulmonary Arterial Compliance in Systemic Lupus Erythematosus Patients with Pulmonary Arterial Hypertension. *Hypertension* (2020) 76(4):1161-8. Epub 2020/08/11. doi: 10.1161/HYPERTENSIONAHA.120.15682.

28. Guo X, Lai J, Wang H, Tian Z, Wang Q, Zhao J, et al. Predictive Value of Non-Invasive Right Ventricle to Pulmonary Circulation Coupling in Systemic Lupus Erythematosus Patients with Pulmonary Arterial Hypertension. *Eur Heart J Cardiovasc Imaging* (2021) 22(1):111-8. Epub 2019/12/25. doi: 10.1093/ehjci/jez311.

29. Haddad F, Spruijt OA, Denault AY, Mercier O, Brunner N, Furman D, et al. Right Heart Score for Predicting Outcome in Idiopathic, Familial, or Drug- and Toxin-Associated Pulmonary Arterial Hypertension. *JACC Cardiovasc Imaging* (2015) 8(6):627-38. Epub 2015/05/20. doi: 10.1016/j.jcmg.2014.12.029.

30. Hsu CH, Glassner C, Foreman AJ, Agarwal R, Benza RJ, Frantz RP, et al. Treadmill Testing Improves Survival Prediction Models in Pulmonary Arterial Hypertension. *Am Heart J* (2011) 162(6):1011-7. Epub 2011/12/06. doi: 10.1016/j.ahj.2011.09.015.

31. Humbert M, Sitbon O, Yaici A, Montani D, O'Callaghan DS, Jais X, et al. Survival in Incident and Prevalent Cohorts of Patients with Pulmonary Arterial Hypertension. *Eur Respir J* (2010) 36(3):549-55. Epub 2010/06/22. doi: 10.1183/09031936.00057010.

32. Kawamukai M, Hashimoto A, Koyama M, Nagano N, Nishida J, Mochizuki A, et al. Risk Classification of Pulmonary Arterial Hypertension by Echocardiographic Combined Assessment of Pulmonary Vascular Resistance and Right Ventricular Function. *Heart Vessels* (2019) 34(11):1789-800. Epub 2019/05/24. doi: 10.1007/s00380-019-01429-7.

33. Kempny A, Hjortshøj CS, Gu H, Li W, Opotowsky AR, Landzberg MJ, et al. Predictors of Death in Contemporary Adult Patients with Eisenmenger Syndrome: A Multicenter Study. *Circulation* (2017) 135(15):1432-40.

34. Lai J, Zhao J, Li K, Qin X, Wang H, Tian Z, et al. Right Ventricle to Pulmonary Artery Coupling Predicts the Risk Stratification in Patients with Systemic Sclerosis-Associated Pulmonary Arterial Hypertension. *Front Cardiovasc Med* (2022) 9:872795.

35. Law MA, Grifka RG, Mullins CE, Nihill MR. Atrial Septostomy Improves Survival in Select Patients with Pulmonary Hypertension. *Am Heart J* (2007) 153(5):779-84. Epub 2007/04/25. doi: 10.1016/j.ahj.2007.02.019.

36. Lewis RA, Armstrong I, Bergbaum C, Brewis MJ, Cannon J, Charalampopoulos A, et al. Emphasis-10 Health-Related Quality of Life Score Predicts Outcomes in Patients with Idiopathic and Connective Tissue Disease-Associated Pulmonary Arterial Hypertension: Results from a Uk Multicentre Study. *Eur Respir J* (2021) 57(2). Epub 2020/07/08. doi: 10.1183/13993003.00124-2020.

37. Ling Y, Johnson MK, Kiely DG, Condliffe R, Elliot CA, Gibbs JS, et al. Changing Demographics, Epidemiology, and Survival of Incident Pulmonary Arterial Hypertension: Results from the Pulmonary Hypertension Registry of the United Kingdom and Ireland. *Am J Respir Crit Care Med* (2012) 186(8):790-6. Epub 2012/07/17. doi: 10.1164/rccm.201203-0383OC.

38. Liu BY, Wu WC, Zeng QX, Liu ZH, Niu LL, Tian Y, et al. The Value of Three-Dimensional Echocardiography in Risk Stratification in Pulmonary Arterial Hypertension: A Cross-Sectional Study. *Int J Cardiovasc Imaging* (2020) 36(4):577-84. Epub 2019/12/20. doi: 10.1007/s10554-019-01743-1.

39. Mazimba S, Mejia-Lopez E, Black G, Kennedy JL, Bergin J, Tallaj JA, et al. Diastolic Pulmonary Gradient Predicts Outcomes in Group 1 Pulmonary Hypertension (Analysis of the Nih Primary Pulmonary Hypertension Registry). *Respir Med* (2016) 119:81-6. Epub 2016/10/04. doi: 10.1016/j.rmed.2016.08.024.

40. Mazimba S, Welch TS, Mwansa H, Breathett KK, Kennedy JLW, Mihalek AD, et al. Haemodynamically Derived Pulmonary Artery Pulsatility Index Predicts Mortality in Pulmonary Arterial Hypertension. *Heart Lung Circ* (2019) 28(5):752-60. Epub 2018/05/12. doi: 10.1016/j.hlc.2018.04.280.

41. Mwansa H, Bilchick KC, Parker AM, Harding W, Ruth B, Kennedy JLW, et al. Decreased Pulmonary Arterial Proportional Pulse Pressure Is Associated with Increased Mortality in Group 1 Pulmonary Hypertension. *Clinical Cardiology* (2017) 40(11):988-92. doi: 10.1002/clc.22752.

42. Nickel N, Golpon H, Greer M, Knudsen L, Olsson K, Westerkamp V, et al. The Prognostic Impact of Follow-up Assessments in Patients with Idiopathic Pulmonary Arterial Hypertension. *Eur Respir J* (2012) 39(3):589-96. Epub 2011/09/03. doi: 10.1183/09031936.00092311.

43. Prins KW, Kalra R, Rose L, Assad TR, Archer SL, Bajaj NS, et al. Hypochloremia Is a Noninvasive Predictor of Mortality in Pulmonary Arterial Hypertension. *Journal of the American Heart Association* (2020) 9(5):e015221. doi: ARTN e015221

10.1161/JAHA.119.015221.

44. Qaderi V, Weimann J, Harbaum L, Schrage BN, Knappe D, Hennigs JK, et al. Non-Invasive Risk Prediction Based on Right Ventricular Function in Patients with Pulmonary Arterial Hypertension. *J Clin Med* (2021) 10(21). Epub 2021/11/14. doi: 10.3390/jcm10215130.

45. Rehman MB, Garcia R, Christiaens L, Larrieu-Ardilouze E, Howard LS, Nihoyannopoulos P. Power of Resting Echocardiographic Measurements to Classify Pulmonary Hypertension Patients According to European Society of Cardiology Exercise Testing Risk Stratification Cut-Offs. *Int J Cardiol* (2018) 257:291-7. Epub 2018/01/24. doi: 10.1016/j.ijcard.2018.01.042.

46. Rhodes CJ, Wharton J, Ghataorhe P, Watson G, Girerd B, Howard LS, et al. Plasma Proteome Analysis in Patients with Pulmonary Arterial Hypertension: An Observational Cohort Study. *Lancet Respir Med* (2017) 5(9):717-26. Epub 2017/06/19. doi: 10.1016/S2213-2600(17)30161-3.

47. Ruth BK, Bilchick KC, Mysore MM, Mwansa H, Harding WC, Kwon Y, et al. Increased Pulmonary-Systemic Pulse Pressure Ratio Is Associated with Increased Mortality in Group 1 Pulmonary Hypertension. *Heart Lung Circ* (2019) 28(7):1059-66. Epub 2018/07/15. doi: 10.1016/j.hlc.2018.05.199.

48. Siddiqui I, Rajagopal S, Brucker A, Chiswell K, Christopher B, Alenezi F, et al. Clinical and Echocardiographic Predictors of Outcomes in Patients with Pulmonary Hypertension. *Am J Cardiol* (2018) 122(5):872-8. Epub 2018/08/11. doi: 10.1016/j.amjcard.2018.05.019.

49. Sandoval J, Bauerle O, Palomar A, Gomez A, Martinez-Guerra ML, Beltran M, et al. Survival in Primary Pulmonary Hypertension. Validation of a Prognostic Equation. *Circulation* (1994) 89(4):1733-44. Epub 1994/04/01. doi: 10.1161/01.cir.89.4.1733.

50. Thenappan T, Shah SJ, Rich S, Tian L, Archer SL, Gomberg-Maitland M. Survival in Pulmonary Arterial Hypertension: A Reappraisal of the Nih Risk Stratification Equation. *Eur Respir J* (2010) 35(5):1079-87. Epub 2009/12/25. doi: 10.1183/09031936.00072709.

51. Thenappan T, Glassner C, Gomberg-Maitland M. Validation of the Pulmonary Hypertension Connection Equation for Survival Prediction in Pulmonary Arterial Hypertension. *Chest* (2012) 141(3):642-50. Epub 2011/09/03. doi: 10.1378/chest.11-0969.

52. van der Bruggen CE, Handoko ML, Bogaard HJ, Marcus JT, Oosterveer FPT, Meijboom LJ, et al. The Value of Hemodynamic Measurements or Cardiac Mri in the Follow-up of Patients with Idiopathic Pulmonary Arterial Hypertension. *Chest* (2021) 159(4):1575-85. Epub 2020/11/17. doi: 10.1016/j.chest.2020.10.077.

53. Arvanitaki A, Boutsikou M, Anthi A, Apostolopoulou S, Avgeropoulou A, Demerouti E, et al. Epidemiology and Initial Management of Pulmonary Arterial Hypertension: Real-World Data from the Hellenic Pulmonary Hypertension Registry (Hope). *Pulm Circ* (2019) 9(3):2045894019877157. Epub 2019/10/31. doi: 10.1177/2045894019877157.

54. Badagliacca R, Poscia R, Pezzuto B, Papa S, Pesce F, Manzi G, et al. Right Ventricular Concentric Hypertrophy and Clinical Worsening in Idiopathic Pulmonary Arterial Hypertension. *J Heart Lung Transplant* (2016) 35(11):1321-9. Epub 2016/06/01. doi: 10.1016/j.healun.2016.04.006.

55. Badagliacca R, Rischard F, Giudice FL, Howard L, Papa S, Valli G, et al. Incremental Value of Cardiopulmonary Exercise Testing in Intermediate-Risk Pulmonary Arterial Hypertension. *J Heart Lung Transplant* (2022) 41(6):780-90.

56. Bouzina H, Hesselstrand R, Radegran G. Plasma Insulin-Like Growth Factor Binding Protein 1 in Pulmonary Arterial Hypertension. *Scand Cardiovasc J* (2021) 55(1):35-42. Epub 2020/07/01. doi: 10.1080/14017431.2020.1782977.

57. Grignola JC, Domingo E, Lopez-Meseguer M, Trujillo P, Bravo C, Perez-Hoyos S, et al. Pulmonary Arterial Remodeling Is Related to the Risk Stratification and Right Ventricular-Pulmonary Arterial Coupling in Patients with Pulmonary Arterial Hypertension. *Frontiers in Physiology* (2021) 12:631326. doi: ARTN 631326

10.3389/fphys.2021.631326.

58. Hoffmann-Vold AM, Hesselstrand R, Fretheim H, Ueland T, Andreassen AK, Brunborg C, et al. Ccl21 as a Potential Serum Biomarker for Pulmonary Arterial Hypertension in Systemic Sclerosis. *Arthritis Rheumatol* (2018) 70(10):1644-53. Epub 2018/04/25. doi: 10.1002/art.40534.

59. Hrustanovic-Kadic M, Ziegler C, El-Kersh K. Palliative Care Perception in Pulmonary Arterial Hypertension: Evaluating the Interaction of Ppci, Pah-Sympact Questionnaire, and the Reveal 2.0 Risk Score. *Ann Am Thorac Soc* (2021) 18(2):361-4. Epub 2020/09/17. doi: 10.1513/AnnalsATS.202005-552RL.

60. Min J, Badesch D, Chakinala M, Elwing J, Frantz R, Horn E, et al. Prediction of Health-Related Quality of Life and Hospitalization in Pulmonary Arterial Hypertension: The Pulmonary Hypertension Association Registry. *Am J Respir Crit Care Med* (2021) 203(6):761-4. Epub 2020/11/20. doi: 10.1164/rccm.202010-3967LE.

61. Sahay S, Tonelli AR, Selej M, Watson Z, Benza RL. Risk Assessment in Patients with Functional Class Ii Pulmonary Arterial Hypertension: Comparison of Physician Gestalt with Esc/Ers and the Reveal 2.0 Risk Score. *PLoS One* (2020) 15(11):e0241504. Epub 2020/11/12. doi: 10.1371/journal.pone.0241504.

62. Sahay S, Bhatt J, Beshay S, Guha A, Nguyen DT, Graviss EA, et al. E-Reveal Lite 2.0 Scoring for Early Prediction of Disease Progression in Pulmonary Arterial Hypertension. *Pulm Circ* (2022) 12(1):e12026.

63. Scelsi L, Ghio S, Matrone B, Mannucci L, Klersy C, Valaperta S, et al. Galectin-3 Plasma Levels Are Associated with Risk Profiles in Pulmonary Arterial Hypertension. *Diagnostics (Basel)* (2020) 10(11). Epub 2020/10/28. doi: 10.3390/diagnostics10110857.

64. Simons JE, Mann EB, Pierozynski A. Assessment of Risk of Disease Progression in Pulmonary Arterial Hypertension: Insights from an International Survey of Clinical Practice. *Adv Ther* (2019) 36(9):2351-63. Epub 2019/07/18. doi: 10.1007/s12325-019-01030-4.

65. Stolfo D, Albani S, Biondi F, De Luca A, Barbati G, Howard L, et al. Global Right Heart Assessment with Speckle-Tracking Imaging Improves the Risk Prediction of a Validated Scoring System in Pulmonary Arterial Hypertension. *J Am Soc Echocardiogr* (2020) 33(11):1334-44 e2. Epub 2020/08/05. doi: 10.1016/j.echo.2020.05.020.

66. Cornwell WK, McLaughlin VV, Krishnan SM, Rubenfire M. Does the Outcome Justify an Oral-First Treatment Strategy for Management of Pulmonary Arterial Hypertension? *Chest* (2011) 140(3):697-705.

67. Ablonczy L, Ferenci T, Somoskovi O, Osvath R, Reusz GS, Kis E. Prognostic Value of Early Risk Stratification in Pediatric Pulmonary Arterial Hypertension. *Transplant Proc* (2021) 53(5):1439-42. Epub 2021/02/25. doi: 10.1016/j.transproceed.2021.01.047.

68. Vlachou M, Fayed H, Dawson A, Reddecliffe S, Stevenson A, Thomson RJ, et al. Intravenous Prostanoids in Systemic Sclerosis-Associated Pulmonary Arterial Hypertension: A Single-Centre Experience. *Rheumatology (Oxford)* (2022) 61(3):1106-14. Epub 2021/06/19. doi: 10.1093/rheumatology/keab478.

69. Boucly A, Weatherald J, Humbert M, Sitbon O. Risk Assessment in Pulmonary Arterial Hypertension. *Eur Respir J* (2018) 51(3). Epub 2018/03/31. doi: 10.1183/13993003.00279-2018.

70. Clerico A, Giannoni A. Will High-Sensitive Troponin Immunoassays Lead to More Clarity or Confusion in Clinical Practice? *Clin Sci (Lond)* (2010) 119(5):203-5. Epub 2010/05/06. doi: 10.1042/CS20100234.

71. Hjalmarsson C, Radegran G, Kylhammar D, Rundqvist B, Multing J, Nisell MD, et al. Impact of Age and Comorbidity on Risk Stratification in Idiopathic Pulmonary Arterial Hypertension. *Eur Respir J* (2018) 51(5). Epub 2018/04/07. doi: 10.1183/13993003.02310-2017.

72. Kissoon N. Treatment of Persistent Pulmonary Hypertension of the Newborn (Pphn) Is in Its Infancy. *J Crit Care* (2006) 21(2):223. Epub 2006/06/14. doi: 10.1016/j.jcrc.2005.12.002.

73. Olsson KM, Richter MJ, Kamp JC, Gall H, Ghofrani HA, Fuge J, et al. Refined Risk Stratification in Pulmonary Arterial Hypertension and Timing of Lung Transplantation. *Eur Respir J* (2022). Epub 2022/02/12. doi: 10.1183/13993003.03087-2021.

74. Packer M. Is It Ethical to Administer Vasodilator Drugs to Patients with Primary Pulmonary Hypertension? *Chest* (1989) 95(6):1173-5.

75. Rubin LJ, Block PC. Evaluating and Treating Pulmonary Hypertension. *ACC Cardiosource Review Journal* (2006) 15(8):68-73.

76. Smith PG, Blumer J. No Good--or Not? *Crit Care Med* (1999) 27(6):1059-60.

77. Valentin S, Maurac A, Sitbon O, Beurnier A, Gomez E, Guillaumot A, et al. Outcomes of Patients with Decreased Arterial Oxyhaemoglobin Saturation on Pulmonary Arterial Hypertension Drugs. *Eur Respir J* (2021) 58(5). Epub 2021/04/21. doi: 10.1183/13993003.04066-2020.

78. Warnes CA, Block PC. Pulmonary Hypertension and Pregnancy. *Journal of the American College of Cardiology* (2006) 47(11):Cs2-Cs6.

79. Wilson M, Keeley J, Kingman M, Wang JJ, Rogers F. Current Clinical Utilization of Risk Assessment Tools in Pulmonary Arterial Hypertension: A Descriptive Survey of Facilitation Strategies, Patterns, and Barriers to Use in the United States. *Pulmonary Circulation* (2020) 10(3):2045894020950186. doi: Artn 2045894020950186

10.1177/2045894020950186.

80. Xiong W, Xu M, Zhao Y, Pudasaini B, Han F, Guo X, et al. Express: A Modified Risk Assessment Score in the Prognostic Evaluation of One-Year Survival Rate of Pulmonary Arterial Hypertension. *Pulm Circ* (2018):2045894018797049. Epub 2018/08/21. doi: 10.1177/2045894018797049.
