# Supplementary material for: Risk stratification in adult and pediatric pulmonary arterial hypertension: A systematic review
Source: Front Cardiovasc Med. 2022 Nov 10;9:1035453. doi: 10.3389/fcvm.2022.1035453 (PMC9684185; doi:10.3389/fcvm.2022.1035453)
Supplement: Supplementary file 3 [file Table_3.DOCX]

Supplementary Material

Supplementary Table 3. Studies eligible for inclusion during full text review.

| **First author** | **Journal (abbreviated)** | **Year** | **Population** | **Study type** |
| --- | --- | --- | --- | --- |
| Austin (1) | Am Heart J | 2017 | Adult | Model development |
| Benza (2) | Chest | 2012 | Adult | Model development |
| Benza (3) | Chest | 2019 | Adult | Model development |
| Benza (4) | Chest | 2021 | Adult | Model development |
| Boucly (5) | Eur Respir J | 2017 | Adult | Model development |
| Chiu (6) | Int J Cardiol | 2020 | Adult | Model development |
| Dardi (7) | Open Heart | 2021 | Adult | Model development |
| Ghio (8) | Eur Respir J | 2020 | Adult | Model development |
| Haarman (9) | Am J Respir Crit Care Med | 2019 | Pediatric | Model development |
| Haddad (10) | Chest | 2022 | Adult | Model development |
| Hoeper (11) | Eur Respir J | 2017 | Adult | Model development |
| Hoeper (12) | Eur Respir J | 2021 | Adult | Model development |
| Imai (13) | Can J Physiol Pharmacol | 2020 | Adult | Model development |
| Kylhammar (14) | Eur Heart J | 2018 | Adult | Model development |
| Lee (15) | Eur Respir J | 2012 | Adult | Model development |
| Li (16) | Clin Rheumatol | 2019 | Adult | Model development |
| Mercurio (17) | Eur Respir J | 2018 | Adult | Model development |
| Rhodes (18) | Am J Respir Crit Care Med | 2022 | Adult | Model development |
| Wang (19) | Eur Respir J | 2020 | Adult | Model development |
| Xiong (20) | BMC Pulm Med | 2018 | Adult | Model development |
| Yogeswaran (21) | J Heart Lung Transplant | 2022 | Adult | Model development |
| Anderson (22) | Chest | 2020 | Adult | Model validation |
| Boucly (23) | Eur Respir J | 2021 | Adult | Model validation |
| Chang (24) | J Am Heart Assoc | 2022 | Adult | Model validation |
| Gong (25) | Front Cardiovasc Med | 2021 | Adult | Model validation |
| Hjalmarsson (26) | ERJ Open Res | 2021 | Adult | Model validation |
| Kylhammar (27) | ERJ Open Res | 2021 | Adult | Model validation |
| Mullin (28) | Arthritis Rheumatol | 2019 | Adult | Model validation |
| Qu (29) | Front Med (Lausanne) | 2021 | Adult | Model validation |
| Quan (30) | Respirology | 2022 | Adult | Model validation |
| Sitbon (31) | Eur Respir J | 2015 | Adult | Model validation |
| Vraka (32) | Respiration | 2022 | Adult | Model validation |
| Weatherald (33) | Eur Respir J | 2018 | Adult | Model validation |
| Xanthouli (34) | Respir Res | 2020 | Adult | Model validation |
| Griffiths (35) | Chest | 2021 | Pediatric | Model enhancement |
| Harbaum (36) | Int J Cardiol | 2020 | Adult | Model enhancement |
| Kanwar (37) | Eur Respir J | 2020 | Adult | Model enhancement |
| Lewis (38) | Am J Respir Crit Care Med | 2020 | Adult | Model enhancement |
| Lewis (39) | Ann Am Thorac Soc | 2021 | Adult | Model enhancement |
| Simpson (40) | ERJ Open Res | 2021 | Adult | Model enhancement |
| Vicenzi (41) | PLoS One | 2022 | Adult | Model enhancement |
| Yogeswaran (42) | Pulm Circ | 2020 | Adult | Model enhancement |
| Zelt (43) | J Heart Lung Transplant | 2020 | Adult | Model enhancement |
| Aldalaan (44) | Pulm Circ | 2022 | Adult | Serial risk stratification |
| Badagliacca (45) | JACC Cardiovasc Imaging | 2020 | Adult | Serial risk stratification |
| Badagliacca (46) | Am J Respir Crit Care Med | 2021 | Adult | Serial risk stratification |
| Barnikel (47) | Pulm Circ | 2019 | Adult | Serial risk stratification |
| Bartenstein (48) | Respiration | 2018 | Adult | Serial risk stratification |
| Bartolome (49) | Chest | 2018 | Adult | Serial risk stratification |
| Benza (50) | J Heart Lung Transplant | 2015 | Adult | Serial risk stratification |
| Benza (51) | J Heart Lung Transplant | 2018 | Adult | Serial risk stratification |
| Benza (52) | International Journal of Cardiology | 2020 | Adult | Serial risk stratification |
| Benza (53) | Int J Cardiol | 2021 | Adult | Serial risk stratification |
| Benza (54) | J Heart Lung Transplant | 2022 | Adult | Serial risk stratification |
| Bouzina (55) | ESC Heart Fail | 2021 | Adult | Serial risk stratification |
| D'Alto (56) | J Heart Lung Transplant | 2020 | Adult | Serial risk stratification |
| Durongpisitkul (57) | JRSM Cardiovascular Disease | 2021 | Adult & pediatric | Serial risk stratification |
| Enderby (58) | Ther Adv Chronic Dis | 2018 | Adult | Serial risk stratification |
| Escribano-Subias (59) | BMC Pulm Med | 2020 | Adult | Serial risk stratification |
| Frost (60) | J Heart Lung Transplant | 2018 | Adult | Serial risk stratification |
| Hjalmarsson (61) | Pulm Circ | 2020 | Adult | Serial risk stratification |
| Hoeper (62) | Eur Respir J | 2018 | Adult | Serial risk stratification |
| Hoeper (63) | J Heart Lung Transplant | 2022 | Adult | Serial risk stratification |
| Humbert (64) | Eur Respir J | 2019 | Adult | Serial risk stratification |
| Kramer (65) | Journal of Cachexia, Sarcopenia and Muscle | 2021 | Adult | Serial risk stratification |
| Olsson (66) | J Heart Lung Transplant | 2019 | Adult | Serial risk stratification |
| Scott (67) | Am J Respir Crit Care Med | 2021 | Adult | Serial risk stratification |
| Sitbon (68) | J Heart Lung Transplant | 2020 | Adult | Serial risk stratification |
| Tamura (69) | BMC Pulm Med | 2021 | Adult | Serial risk stratification |
| Tonelli (70) | Pulm Circ | 2020 | Adult | Serial risk stratification |
| Vanderpool (71) | Chest | 2021 | Adult | Serial risk stratification |
| White (72) | Am J Respir Crit Care Med | 2020 | Adult | Serial risk stratification |
| Yoshida (73) | Nagoya J Med Sci | 2021 | Adult | Serial risk stratification |
| Zhao (74) | Front Cardiovasc Med | 2021 | Adult | Serial risk stratification |

# Supplementary references

1. Austin C, Burger C, Kane G, Safford R, Blackshear J, Ung R, et al. High-Risk Echocardiographic Features Predict Mortality in Pulmonary Arterial Hypertension. *Am Heart J* (2017) 189:167-76. Epub 2017/06/20. doi: 10.1016/j.ahj.2017.04.013.

2. Benza RL, Gomberg-Maitland M, Miller DP, Frost A, Frantz RP, Foreman AJ, et al. The Reveal Registry Risk Score Calculator in Patients Newly Diagnosed with Pulmonary Arterial Hypertension. *Chest* (2012) 141(2):354-62. Epub 2011/06/18. doi: 10.1378/chest.11-0676.

3. Benza RL, Gomberg-Maitland M, Elliott CG, Farber HW, Foreman AJ, Frost AE, et al. Predicting Survival in Patients with Pulmonary Arterial Hypertension: The Reveal Risk Score Calculator 2.0 and Comparison with Esc/Ers-Based Risk Assessment Strategies. *Chest* (2019) 156(2):323-37. Epub 2019/02/18. doi: 10.1016/j.chest.2019.02.004.

4. Benza RL, Kanwar MK, Raina A, Scott JV, Zhao CL, Selej M, et al. Development and Validation of an Abridged Version of the Reveal 2.0 Risk Score Calculator, Reveal Lite 2, for Use in Patients with Pulmonary Arterial Hypertension. *Chest* (2021) 159(1):337-46. doi: 10.1016/j.chest.2020.08.2069.

5. Boucly A, Weatherald J, Savale L, Jais X, Cottin V, Prevot G, et al. Risk Assessment, Prognosis and Guideline Implementation in Pulmonary Arterial Hypertension. *Eur Respir J* (2017) 50(2). Epub 2017/08/05. doi: 10.1183/13993003.00889-2017.

6. Chiu SN, Weng KP, Lin MC, Wang JN, Hwang BT, Dai ZK, et al. Congenital Heart Disease with Pulmonary Artery Hypertension in an Asian Cohort-Initial Report from Tachyon (Taiwan Congenital Heart Disease Associated with Pulmonary Arterial Hypertension) Registry. *Int J Cardiol* (2020) 317:49-55. Epub 2020/06/12. doi: 10.1016/j.ijcard.2020.05.086.

7. Dardi F, Manes A, Guarino D, Zuffa E, De Lorenzis A, Magnani I, et al. A Pragmatic Approach to Risk Assessment in Pulmonary Arterial Hypertension Using the 2015 European Society of Cardiology/European Respiratory Society Guidelines. *Open Heart* (2021) 8(2). doi: ARTN e001725

10.1136/openhrt-2021-001725.

8. Ghio S, Mercurio V, Fortuni F, Forfia PR, Gall H, Ghofrani A, et al. A Comprehensive Echocardiographic Method for Risk Stratification in Pulmonary Arterial Hypertension. *Eur Respir J* (2020) 56(3). Epub 2020/05/21. doi: 10.1183/13993003.00513-2020.

9. Haarman MG, Douwes JM, Ploegstra MJ, Roofthooft MTR, Vissia-Kazemier TR, Hillege HL, et al. The Clinical Value of Proposed Risk Stratification Tools in Pediatric Pulmonary Arterial Hypertension. *Am J Respir Crit Care Med* (2019) 200(10):1312-5. Epub 2019/07/13. doi: 10.1164/rccm.201902-0266LE.

10. Haddad F, Contrepois K, Amsallem M, Denault AY, Bernardo RJ, Jha A, et al. The Right Heart Network and Risk Stratification in Pulmonary Arterial Hypertension. *Chest* (2022) 161(5):1347-59. Epub 2021/11/15. doi: 10.1016/j.chest.2021.10.045.

11. Hoeper MM, Kramer T, Pan Z, Eichstaedt CA, Spiesshoefer J, Benjamin N, et al. Mortality in Pulmonary Arterial Hypertension: Prediction by the 2015 European Pulmonary Hypertension Guidelines Risk Stratification Model. *Eur Respir J* (2017) 50(2). Epub 2017/08/05. doi: 10.1183/13993003.00740-2017.

12. Hoeper MM, Pausch C, Olsson KM, Huscher D, Pittrow D, Grunig E, et al. Compera 2.0: A Refined 4-Strata Risk Assessment Model for Pulmonary Arterial Hypertension. *Eur Respir J* (2021). Epub 2021/11/06. doi: 10.1183/13993003.02311-2021.

13. Imai R, Adachi S, Yoshida M, Shimokata S, Nakano Y, Okumura N, et al. Single-Center Prognostic Validation of the Risk Assessment of the 2015 Esc/Ers Guidelines in Patients with Pulmonary Arterial Hypertension in Japan. *Can J Physiol Pharmacol* (2020) 98(9):653-8. Epub 2020/09/15. doi: 10.1139/cjpp-2019-0640.

14. Kylhammar D, Kjellstrom B, Hjalmarsson C, Jansson K, Nisell M, Soderberg S, et al. A Comprehensive Risk Stratification at Early Follow-up Determines Prognosis in Pulmonary Arterial Hypertension. *European Heart Journal* (2018) 39(47):4175-81. doi: 10.1093/eurheartj/ehx257.

15. Lee WT, Ling Y, Sheares KK, Pepke-Zaba J, Peacock AJ, Johnson MK. Predicting Survival in Pulmonary Arterial Hypertension in the Uk. *Eur Respir J* (2012) 40(3):604-11. Epub 2012/05/05. doi: 10.1183/09031936.00196611.

16. Li X, Sun X, Huang Y, Wang Y, Yang X, Wang J, et al. Simplified Risk Stratification for Pulmonary Arterial Hypertension Associated with Connective Tissue Disease. *Clin Rheumatol* (2019) 38(12):3619-26. Epub 2019/08/07. doi: 10.1007/s10067-019-04690-3.

17. Mercurio V, Diab N, Peloquin G, Housten-Harris T, Damico R, Kolb TM, et al. Risk Assessment in Scleroderma Patients with Newly Diagnosed Pulmonary Arterial Hypertension: Application of the Esc/Ers Risk Prediction Model. *Eur Respir J* (2018) 52(4). Epub 2018/09/29. doi: 10.1183/13993003.00497-2018.

18. Rhodes CJ, Wharton J, Swietlik EM, Harbaum L, Girerd B, Coghlan JG, et al. Using the Plasma Proteome for Risk Stratifying Patients with Pulmonary Arterial Hypertension. *Am J Respir Crit Care Med* (2022) 205(9):1102-11. Epub 2022/01/27. doi: 10.1164/rccm.202105-1118OC.

19. Wang J, Li M, Wang Q, Zhang X, Qian J, Zhao J, et al. Pulmonary Arterial Hypertension Associated with Primary Sjogren's Syndrome: A Multicentre Cohort Study from China. *Eur Respir J* (2020) 56(5). Epub 2020/07/04. doi: 10.1183/13993003.02157-2019.

20. Xiong W, Zhao Y, Xu M, Pudasaini B, Guo X, Liu J. A Modified Risk Score in One-Year Survival Rate Assessment of Group 1 Pulmonary Arterial Hypertension. *BMC Pulm Med* (2018) 18(1):161. Epub 2018/10/18. doi: 10.1186/s12890-018-0712-7.

21. Yogeswaran A, Tello K, Lund J, Klose H, Harbaum L, Sommer N, et al. Risk Assessment in Pulmonary Hypertension Based on Routinely Measured Laboratory Parameters. *J Heart Lung Transplant* (2022) 41(3):400-10. Epub 2021/12/04. doi: 10.1016/j.healun.2021.10.018.

22. Anderson JJ, Lau EM, Lavender M, Benza R, Celermajer DS, Collins N, et al. Retrospective Validation of the Reveal 2.0 Risk Score with the Australian and New Zealand Pulmonary Hypertension Registry Cohort. *Chest* (2020) 157(1):162-72. Epub 2019/09/30. doi: 10.1016/j.chest.2019.08.2203.

23. Boucly A, Weatherald J, Savale L, de Groote P, Cottin V, Prevot G, et al. External Validation of a Refined 4-Strata Risk Assessment Score from the French Pulmonary Hypertension Registry. *Eur Respir J* (2021). Epub 2021/11/06. doi: 10.1183/13993003.02419-2021.

24. Chang KY, Duval S, Badesch DB, Bull TM, Chakinala MM, De Marco T, et al. Mortality in Pulmonary Arterial Hypertension in the Modern Era: Early Insights from the Pulmonary Hypertension Association Registry. *J Am Heart Assoc* (2022) 11(9):e024969.

25. Gong SG, Wu WH, Li C, Zhao QH, Jiang R, Luo CJ, et al. Validity of the Esc Risk Assessment in Idiopathic Pulmonary Arterial Hypertension in China. *Front Cardiovasc Med* (2021) 8:745578. Epub 2021/12/10. doi: 10.3389/fcvm.2021.745578.

26. Hjalmarsson C, Kjellstrom B, Jansson K, Nisell M, Kylhammar D, Kavianipour M, et al. Early Risk Prediction in Idiopathic Versus Connective Tissue Disease-Associated Pulmonary Arterial Hypertension: Call for a Refined Assessment. *ERJ Open Res* (2021) 7(3). Epub 2021/08/06. doi: 10.1183/23120541.00854-2020.

27. Kylhammar D, Hjalmarsson C, Hesselstrand R, Jansson K, Kavianipour M, Kjellstrom B, et al. Predicting Mortality During Long-Term Follow-up in Pulmonary Arterial Hypertension. *ERJ Open Res* (2021) 7(2). Epub 2021/06/05. doi: 10.1183/23120541.00837-2020.

28. Mullin CJ, Khair RM, Damico RL, Kolb TM, Hummers LK, Hassoun PM, et al. Validation of the Reveal Prognostic Equation and Risk Score Calculator in Incident Systemic Sclerosis-Associated Pulmonary Arterial Hypertension. *Arthritis Rheumatol* (2019) 71(10):1691-700. Epub 2019/05/09. doi: 10.1002/art.40918.

29. Qu J, Li M, Zeng X, Zhang X, Wei W, Zuo X, et al. Validation of the Reveal Prognostic Models in Systemic Lupus Erythematosus-Associated Pulmonary Arterial Hypertension. *Front Med (Lausanne)* (2021) 8:618486. Epub 2021/03/23. doi: 10.3389/fmed.2021.618486.

30. Quan R, Zhang G, Yu Z, Zhang C, Yang Z, Tian H, et al. Characteristics, Goal-Oriented Treatments and Survival of Pulmonary Arterial Hypertension in China: Insights from a National Multicentre Prospective Registry. *Respirology* (2022). Epub 2022/03/17. doi: 10.1111/resp.14247.

31. Sitbon O, Benza RL, Badesch DB, Barst RJ, Elliott CG, Gressin V, et al. Validation of Two Predictive Models for Survival in Pulmonary Arterial Hypertension. *Eur Respir J* (2015) 46(1):152-64. Epub 2015/04/04. doi: 10.1183/09031936.00004414.

32. Vraka A, Yerly P, Aubert JD. Comparison of Risk Stratification Scores in Pulmonary Arterial Hypertension: A Monocentric Retrospective Study at Lausanne University Hospital. *Respiration* (2022):1-12. doi: 10.1159/000520886.

33. Weatherald J, Boucly A, Launay D, Cottin V, Prevot G, Bourlier D, et al. Haemodynamics and Serial Risk Assessment in Systemic Sclerosis Associated Pulmonary Arterial Hypertension. *Eur Respir J* (2018) 52(4). Epub 2018/09/14. doi: 10.1183/13993003.00678-2018.

34. Xanthouli P, Koegler M, Marra AM, Benjamin N, Fischer L, Eichstaedt CA, et al. Risk Stratification and Prognostic Factors in Patients with Pulmonary Arterial Hypertension and Comorbidities a Cross-Sectional Cohort Study with Survival Follow-Up. *Respir Res* (2020) 21(1):127. Epub 2020/05/26. doi: 10.1186/s12931-020-01393-1.

35. Griffiths M, Yang J, Simpson CE, Vaidya D, Nies M, Brandal S, et al. St2 Is a Biomarker of Pediatric Pulmonary Arterial Hypertension Severity and Clinical Worsening. *Chest* (2021) 160(1):297-306. Epub 2021/02/21. doi: 10.1016/j.chest.2021.01.085.

36. Harbaum L, Fuge J, Kamp JC, Hennigs JK, Simon M, Sinning C, et al. Blood Carbon Dioxide Tension and Risk in Pulmonary Arterial Hypertension. *Int J Cardiol* (2020) 318:131-7. Epub 2020/07/08. doi: 10.1016/j.ijcard.2020.06.069.

37. Kanwar MK, Gomberg-Maitland M, Hoeper M, Pausch C, Pittrow D, Strange G, et al. Risk Stratification in Pulmonary Arterial Hypertension Using Bayesian Analysis. *Eur Respir J* (2020) 56(2).

38. Lewis RA, Johns CS, Cogliano M, Capener D, Tubman E, Elliot CA, et al. Identification of Cardiac Magnetic Resonance Imaging Thresholds for Risk Stratification in Pulmonary Arterial Hypertension. *Am J Respir Crit Care Med* (2020) 201(4):458-68. Epub 2019/10/28. doi: 10.1164/rccm.201909-1771OC.

39. Lewis RA, Billings CG, Hurdman JA, Smith IA, Austin M, Armstrong IJ, et al. Maximal Exercise Testing Using the Incremental Shuttle Walking Test Can Be Used to Risk-Stratify Patients with Pulmonary Arterial Hypertension. *Annals of the American Thoracic Society* (2021) 18(1):34-43. doi: 10.1513/AnnalsATS.202005-423OC.

40. Simpson CE, Griffiths M, Yang J, Nies MK, Vaidya RD, Brandal S, et al. The Angiostatic Peptide Endostatin Enhances Mortality Risk Prediction in Pulmonary Arterial Hypertension. *ERJ Open Res* (2021) 7(4). Epub 2021/10/16. doi: 10.1183/23120541.00378-2021.

41. Vicenzi M, Caravita S, Rota I, Casella R, Deboeck G, Beretta L, et al. The Added Value of Right Ventricular Function Normalized for Afterload to Improve Risk Stratification of Patients with Pulmonary Arterial Hypertension. *PLoS One* (2022) 17(5):e0265059.

42. Yogeswaran A, Richter MJ, Sommer N, Ghofrani HA, Seeger W, Tello K, et al. Advanced Risk Stratification of Intermediate Risk Group in Pulmonary Arterial Hypertension. *Pulm Circ* (2020) 10(4):2045894020961739. Epub 2020/10/23. doi: 10.1177/2045894020961739.

43. Zelt JGE, Hossain A, Sun LY, Mehta S, Chandy G, Davies RA, et al. Incorporation of Renal Function in Mortality Risk Assessment for Pulmonary Arterial Hypertension. *J Heart Lung Transplant* (2020) 39(7):675-85. Epub 2020/04/28. doi: 10.1016/j.healun.2020.03.026.

44. Aldalaan AM, Saleemi SA, Weheba I, Abdelsayed A, Aleid MM, Alzubi F, et al. Prospective Clinical Assessment of Patients with Pulmonary Arterial Hypertension Switched from Bosentan to Macitentan (Potent). *Pulm Circ* (2022) 12(2):e12083.

45. Badagliacca R, Papa S, Manzi G, Miotti C, Luongo F, Sciomer S, et al. Usefulness of Adding Echocardiography of the Right Heart to Risk-Assessment Scores in Prostanoid-Treated Pulmonary Arterial Hypertension. *JACC Cardiovasc Imaging* (2020) 13(9):2054-6. Epub 2020/06/22. doi: 10.1016/j.jcmg.2020.04.005.

46. Badagliacca R, D'Alto M, Ghio S, Argiento P, Bellomo V, Brunetti ND, et al. Risk Reduction and Hemodynamics with Initial Combination Therapy in Pulmonary Arterial Hypertension. *Am J Respir Crit Care Med* (2021) 203(4):484-92. Epub 2020/08/29. doi: 10.1164/rccm.202004-1006OC.

47. Barnikel M, Kneidinger N, Klenner F, Waelde A, Arnold P, Sonneck T, et al. Real-Life Data on Selexipag for the Treatment of Pulmonary Hypertension. *Pulm Circ* (2019) 9(1):2045894019832199. Epub 2019/02/05. doi: 10.1177/2045894019832199.

48. Bartenstein P, Saxer S, Appenzeller P, Lichtblau M, Schwarz EI, Ulrich S. Risk Factor Profiles Achieved with Medical Therapy in Prevalent Patients with Pulmonary Arterial and Distal Chronic Thromboembolic Pulmonary Hypertension. *Respiration* (2018) 96(2):127-37. Epub 2018/04/12. doi: 10.1159/000488000.

49. Bartolome SD, Sood N, Shah TG, Styrvoky K, Torres F, Chin KM. Mortality in Patients with Pulmonary Arterial Hypertension Treated with Continuous Prostanoids. *Chest* (2018) 154(3):532-40. Epub 2018/04/22. doi: 10.1016/j.chest.2018.03.050.

50. Benza RL, Miller DP, Foreman AJ, Frost AE, Badesch DB, Benton WW, et al. Prognostic Implications of Serial Risk Score Assessments in Patients with Pulmonary Arterial Hypertension: A Registry to Evaluate Early and Long-Term Pulmonary Arterial Hypertension Disease Management (Reveal) Analysis. *J Heart Lung Transplant* (2015) 34(3):356-61. Epub 2014/12/03. doi: 10.1016/j.healun.2014.09.016.

51. Benza RL, Farber HW, Frost A, Ghofrani HA, Gomez-Sanchez MA, Langleben D, et al. Reveal Risk Scores Applied to Riociguat-Treated Patients in Patent-2: Impact of Changes in Risk Score on Survival. *J Heart Lung Transplant* (2018) 37(4):513-9. Epub 2017/12/11. doi: 10.1016/j.healun.2017.11.006.

52. Benza RL, Corris PA, Klinger JR, Langleben D, Naeije R, Simonneau G, et al. Identifying Potential Parameters Associated with Response to Switching from a Pde5i to Riociguat in Respite. *Int J Cardiol* (2020) 317:188-92. Epub 2020/05/29. doi: 10.1016/j.ijcard.2020.05.044.

53. Benza RL, Farber HW, Frost AE, Ghofrani HA, Corris PA, Lambelet M, et al. Application of the Reveal Risk Score Calculator 2.0 in the Patent Study. *Int J Cardiol* (2021) 332:189-92. Epub 2021/03/22. doi: 10.1016/j.ijcard.2021.03.034.

54. Benza RL, Boucly A, Farber HW, Frost AE, Ghofrani HA, Hoeper MM, et al. Change in Reveal Lite 2 Risk Score Predicts Outcomes in Patients with Pulmonary Arterial Hypertension in the Patent Study. *J Heart Lung Transplant* (2022) 41(3):411-20. Epub 2021/12/02. doi: 10.1016/j.healun.2021.10.013.

55. Bouzina H, Radegran G, Butler O, Hesselstrand R, Hjalmarsson C, Holl K, et al. Longitudinal Changes in Risk Status in Pulmonary Arterial Hypertension. *ESC Heart Fail* (2021) 8(1):680-90. Epub 2020/12/12. doi: 10.1002/ehf2.13162.

56. D'Alto M, Badagliacca R, Lo Giudice F, Argiento P, Casu G, Corda M, et al. Hemodynamics and Risk Assessment 2 Years after the Initiation of Upfront Ambrisentantadalafil in Pulmonary Arterial Hypertension. *J Heart Lung Transplant* (2020) 39(12):1389-97. Epub 2020/09/17. doi: 10.1016/j.healun.2020.08.016.

57. Durongpisitkul K, Chungsomprasong P, Vijarnsorn C, Chanthong P, Kanjanauthai S, Soongswang J. Improved Low-Risk Criteria Scores for Combination Therapy of Sildenafil and Generic Bosentan in Patients with Congenital Heart Disease with Severe Pulmonary Hypertension: A Prospective Open Label Study. *JRSM Cardiovasc Dis* (2021) 10:2048004020982213. Epub 2021/02/23. doi: 10.1177/2048004020982213.

58. Enderby CY, Burger C. Tolerability and Clinical Efficacy of Inhaled Treprostinil in Patients with Group 1 Pulmonary Arterial Hypertension. *Ther Adv Chronic Dis* (2018) 9(9):171-7. Epub 2018/09/06. doi: 10.1177/2040622318779749.

59. Escribano-Subias P, Lopez R, Almenar L, Lazaro M, Forn I, Torrent A, et al. Changes in Reveal Risk Score in Patients with Pulmonary Arterial Hypertension Treated with Macitentan in Clinical Practice: Results from the Pracma Study. *BMC Pulm Med* (2020) 20(1):154. Epub 2020/06/04. doi: 10.1186/s12890-020-01197-5.

60. Frost AE, Hoeper MM, Barbera JA, Vachiery JL, Blair C, Langley J, et al. Risk-Stratified Outcomes with Initial Combination Therapy in Pulmonary Arterial Hypertension: Application of the Reveal Risk Score. *J Heart Lung Transplant* (2018) 37(12):1410-7. Epub 2018/09/14. doi: 10.1016/j.healun.2018.07.001.

61. Hjalmarsson C, Butler O, Hesselstrand R, Holl K, Jansson K, Klok R, et al. Poor Outcome of Patients with Pulmonary Arterial Hypertension with Insufficient Response to Phosphodiesterase-5 Inhibitors Alone or in Combination with Other Specific Therapy: A Registry-Based Study. *Pulm Circ* (2020) 10(3):2045894020958557. Epub 2020/10/17. doi: 10.1177/2045894020958557.

62. Hoeper MM, Pittrow D, Opitz C, Gibbs JSR, Rosenkranz S, Grunig E, et al. Risk Assessment in Pulmonary Arterial Hypertension. *Eur Respir J* (2018) 51(3). Epub 2018/03/31. doi: 10.1183/13993003.02606-2017.

63. Hoeper MM, Pausch C, Olsson KM, Huscher D, Pittrow D, Grünig E, et al. Prognostic Value of Improvement Endpoints in Pulmonary Arterial Hypertension Trials: A Compera Analysis. *J Heart Lung Transplant* (2022).

64. Humbert M, Farber HW, Ghofrani HA, Benza RL, Busse D, Meier C, et al. Risk Assessment in Pulmonary Arterial Hypertension and Chronic Thromboembolic Pulmonary Hypertension. *Eur Respir J* (2019) 53(6). Epub 2019/03/30. doi: 10.1183/13993003.02004-2018.

65. Kramer T, Wissmüller M, Natsina K, Gerhardt F, ten Freyhaus H, Dumitrescu D, et al. Ferric Carboxymaltose in Patients with Pulmonary Arterial Hypertension and Iron Deficiency: A Long-Term Study. *Journal of Cachexia, Sarcopenia and Muscle* (2021) 12(6):1501-12.

66. Olsson KM, Richter MJ, Kamp JC, Gall H, Heine A, Ghofrani HA, et al. Intravenous Treprostinil as an Add-on Therapy in Patients with Pulmonary Arterial Hypertension. *J Heart Lung Transplant* (2019) 38(7):748-56. Epub 2019/05/28. doi: 10.1016/j.healun.2019.05.002.

67. Scott JV, Garnett CE, Kanwar MK, Stockbridge NL, Benza RL. Enrichment Benefits of Risk Algorithms for Pulmonary Arterial Hypertension Clinical Trials. *Am J Respir Crit Care Med* (2021) 203(6):726-36. Epub 2020/09/17. doi: 10.1164/rccm.202002-0357OC.

68. Sitbon O, Chin KM, Channick RN, Benza RL, Di Scala L, Gaine S, et al. Risk Assessment in Pulmonary Arterial Hypertension: Insights from the Griphon Study. *J Heart Lung Transplant* (2020) 39(4):300-9. Epub 2020/02/18. doi: 10.1016/j.healun.2019.12.013.

69. Tamura Y, Kumamaru H, Abe K, Satoh T, Miyata H, Ogawa A, et al. Improvements in French Risk Stratification Score Were Correlated with Reductions in Mean Pulmonary Artery Pressure in Pulmonary Arterial Hypertension: A Subanalysis of the Japan Pulmonary Hypertension Registry (Japhr). *Bmc Pulmonary Medicine* (2021) 21(1):28. doi: ARTN 28

10.1186/s12890-021-01398-6.

70. Tonelli AR, Sahay S, Gordon KW, Edwards LD, Allmon AG, Broderick M, et al. Impact of Inhaled Treprostinil on Risk Stratification with Noninvasive Parameters: A Post Hoc Analysis of the Triumph and Beat Studies. *Pulm Circ* (2020) 10(4):2045894020977025. Epub 2021/01/07. doi: 10.1177/2045894020977025.

71. Vanderpool RR, Hunter KS, Insel M, Garcia JGN, Bedrick EJ, Tedford RJ, et al. The Right Ventricular-Pulmonary Arterial Coupling and Diastolic Function Response to Therapy in Pulmonary Arterial Hypertension. *Chest* (2022) 161(4):1048-59. Epub 2021/10/13. doi: 10.1016/j.chest.2021.09.040.

72. White RJ, Jerjes-Sanchez C, Bohns Meyer GM, Pulido T, Sepulveda P, Wang KY, et al. Combination Therapy with Oral Treprostinil for Pulmonary Arterial Hypertension. A Double-Blind Placebo-Controlled Clinical Trial. *Am J Respir Crit Care Med* (2020) 201(6):707-17. Epub 2019/11/26. doi: 10.1164/rccm.201908-1640OC.

73. Yoshida M, Adachi S, Imai R, Shimokata S, Nakano Y, Murohara T, et al. Editors' Choice Differential Effects of Combination Therapy on the Components of the Risk Stratification Table in Patients with Idiopathic or Heritable Pulmonary Arterial Hypertension in a Japanese Population. *Nagoya J Med Sci* (2021) 83(2):321-30. Epub 2021/07/10. doi: 10.18999/nagjms.83.2.321.

74. Zhao QH, Gong SG, Jiang R, Li C, Chen GF, Luo CJ, et al. Echocardiographic Prognosis Relevance of Attenuated Right Heart Remodeling in Idiopathic Pulmonary Arterial Hypertension. *Front Cardiovasc Med* (2021) 8:650848. Epub 2021/05/25. doi: 10.3389/fcvm.2021.650848.
